# Systematic identification of plausible pathways to potential harm via problem formulation for investigational releases of a population suppression gene drive to control the human malaria vector *Anopheles gambiae* in West Africa

**DOI:** 10.1186/s12936-021-03674-6

**Published:** 2021-03-29

**Authors:** John B. Connolly, John D. Mumford, Silke Fuchs, Geoff Turner, Camilla Beech, Ace R. North, Austin Burt

**Affiliations:** 1 Department of Life Sciences, Imperial College London, London, UK; 2 Centre for Environmental Policy, Imperial College London, London, UK; 3 Cambea Consulting Ltd, Reading, UK; 4 Department of Zoology, University of Oxford, Oxford, UK

**Keywords:** Gene drive, Population suppression gene drive, *Anopheles*, Transgenic, Field release, Malaria, Vector control, Problem formulation, Pathways to harm, Environmental risk assessment (ERA)

## Abstract

**Background:**

Population suppression gene drive has been proposed as a strategy for malaria vector control. A CRISPR-Cas9-based transgene homing at the *doublesex* locus (*dsxF*^*CRISPRh*^) has recently been shown to increase rapidly in frequency in, and suppress, caged laboratory populations of the malaria mosquito vector *Anopheles gambiae*. Here, problem formulation, an initial step in environmental risk assessment (ERA), was performed for simulated field releases of the *dsxF*^*CRISPRh*^ transgene in West Africa.

**Methods:**

Building on consultative workshops in Africa that previously identified relevant environmental and health protection goals for ERA of gene drive in malaria vector control, 8 potentially harmful effects from these simulated releases were identified. These were stratified into 46 plausible pathways describing the causal chain of events that would be required for potential harms to occur. Risk hypotheses to interrogate critical steps in each pathway, and an analysis plan involving experiments, modelling and literature review to test each of those risk hypotheses, were developed.

**Results:**

Most potential harms involved increased human (n = 13) or animal (n = 13) disease transmission, emphasizing the importance to subsequent stages of ERA of data on vectorial capacity comparing transgenics to non-transgenics. Although some of the pathways (n = 14) were based on known anatomical alterations in *dsxF*^*CRISPRh*^ homozygotes, many could also be applicable to field releases of a range of other transgenic strains of mosquito (n = 18). In addition to population suppression of target organisms being an accepted outcome for existing vector control programmes, these investigations also revealed that the efficacy of population suppression caused by the *dsxF*^*CRISPRh*^ transgene should itself directly affect most pathways (n = 35).

**Conclusions:**

Modelling will play an essential role in subsequent stages of ERA by clarifying the dynamics of this relationship between population suppression and reduction in exposure to specific potential harms. This analysis represents a comprehensive identification of plausible pathways to potential harm using problem formulation for a specific gene drive transgene and organism, and a transparent communication tool that could inform future regulatory studies, guide subsequent stages of ERA, and stimulate further, broader engagement on the use of population suppression gene drive to control malaria vectors in West Africa.

## Background

The World Health Organization (WHO) estimated in 2019 there were 229 million cases of malaria globally, accounting for 409,000 deaths [1]. Persistent threats from insecticide resistance have created the need for additional, complementary strategies to control the mosquito vectors that are responsible for transmission of disease [2–5]. Based on mathematical modelling studies, the use of gene drive in vectors such as *Anopheles gambiae* has been proposed as one such complementary approach to vector control [6–12].

Recent advances in the development of synthetic gene drives involve the use of a transgene encoding CRISPR-Cas9 endonuclease that is expressed from a germline promoter, coupled with expression of a guide RNA targeting a specific DNA sequence [13–19]. In heterozygous transgenics, the transgene is inserted at the target site of the guide RNA on one chromosome, while the target site on the homologous chromosome is wild type. In the germline of such heterozygotes, Cas9 uses the guide RNA to recognize and cleave the target sequence of the wild-type chromosome. This causes a double-stranded break in the germline at the target sequence, which is repaired using the homologous chromosome containing the transgene as a template, so that the transgene is copied onto the homologous chromosome that had previously been wild type, in a process known as ‘homing’.

Thus, homing converts a proportion of the germline in transgenics to homozygosity so that a greater proportion of gametes contain the transgene, and therefore a greater proportion of offspring will inherit the transgene than would otherwise be the case. Homing, therefore, allows the transgene to spread through a population with super-Mendelian inheritance. This means that, unlike some other genetically modified insect vector control approaches, gene drive transgenes are intended to increase in frequency and persist in the target organism (TO) to achieve lasting impact on disease transmission [20].

‘Population suppression gene drive’ is a potential way to exploit such a homing transgene in order to decrease the density of mosquito vector populations and thus reduce malaria transmission [5, 12, 21]. One method involves exploitation of a haplo-sufficient female fertility gene, mutations of which can cause female sterility when homozygous, but not when heterozygous [6, 12, 16, 17, 21]. The CRISPR-Cas9 system is encoded by a transgene that uses a guide RNA to target such a female fertility gene allowing the transgene to spread at super-Mendelian levels through the population via homing, disrupting the female fertility gene as it does so. As the heterozygous transgenics are fertile, their frequency steadily increases in the population after introduction of the transgene. As heterozygous transgenic females and males begin increasingly to encounter and mate with each another, the proportion of the female population that is homozygous for the transgene increases. However, as they are sterile, the number of viable eggs produced decreases until eventually the population is suppressed [17].

The Target Malaria not-for-profit research consortium has been engaged in the co-development of gene drive as a malaria vector control tool in Africa [22]. Before a population suppression gene drive system undergoes regulatory review for field release, plausible risks to human health and the environment must first be identified and assessed [3, 4, 23–35]. Indeed, James et al. [30] proposed that *“the safety standard for moving an investigational gene drive product from physical confinement to field testing should be a well-reasoned justification that it will do no more harm to human health than wild-type mosquitoes of the same genetic background and no more harm to the ecosystem than other conventional vector control interventions.”*

Problem formulation is a rigorous scientific analysis that defines the overall parameters for an environmental risk assessment (ERA) and facilitates the systematic identification of potential harms or hazards, as well as their routes of exposure, whilst being transparent about the assumptions that have been made during the process (see Table 1 for Glossary of Terms) [33–39]. The WHO has developed a guidance framework for testing genetically modified mosquitoes to ensure that organisms are effective and competitive and that risks are reduced to acceptable levels [40], so that gene drive organisms intended for release should have already been tested for deleterious phenotypes or unacceptable, unintended effects during product development. Thus, ERAs can focus on the introduced genetic construct and the intended outcome. ERAs should specifically address protection goals that are identified from policy, legislative, regulatory and community requirements from the region where the intervention is being considered. Referring to such protection goals, a wide range of potential harms is initially considered in a highly iterative, systematic approach involving a diverse range of expert input. An ERA of the release of a genetically modified organism needs to consider both direct effects on individual organisms that the transgenic itself generates, such as via predation, competition, habitat alteration, hybridization and introduction of new parasites and diseases, and indirect effects such as those on individual organisms in the wider environment without immediate contact with the transgenic (Table 1) [26]. Based on the biological information available on both the transgenic and its parental species, as well as the wider environment into which the transgene will be released, the plausibility of each pathway to potential harm to the identified protection goals is examined by establishing the causal chain of events, sometimes condensed to “pathway to harm” [41]. To determine the cogency of each pathway, risk hypotheses are constructed that can be used to interrogate individual steps in the pathway. Next, an analysis plan is developed that includes both defined measurement endpoints to test risk hypotheses and other potential sources of evidence aimed at reducing identified areas of uncertainty surrounding pathways to each potential harm. Such evidence may be sought from pre-existing literature, modelling, new experimental investigations, previous experiences [35], or any combination thereof. The analysis plan will make important contributions to subsequent steps of hazard, exposure and risk characterization in the ERA. This also allows the identification of hypotheses that can be tested most reliably and efficiently. The potential for harms to occur can be considered negligible where a risk hypothesis can be accepted on the basis of unequivocal evidence and minimal remaining uncertainties surrounding the pathway. Where this is not possible, a number of risk hypotheses must be tested using a ‘weight of evidence’ approach that draws upon several sources of evidence for assessment of the pathway [35, 42].

**Table 1:** Glossary of terms

Term	Definition	References	
Analysis Plan	Describes the evidence and measurement endpoints to be used in the ERA, which can be tiered to prioritize the most informative evidence for decision-making	[80]	
Assessment Endpoint	Explicit expression of environmental or health value to be protected	[37]	
Direct Effect	Effects on individual organisms that the transgenic itself generates, such as via predation, competition, hybridization and introduction of new parasites and diseases.	[26]	
Environmental Risk Assessment (ERA)	Process to identify significant risks to the environment and health, estimating their magnitude and likelihood and defining any risk management required	[25–27, 37]	
Conceptual Model	Environmental and health entities of value and their measurable attributes	[37]	
Ecosystem Services	‘Provisioning services’ such as water, ‘Regulating services’ such as pollination and ‘Supporting services’ such as nutrient recycling, delivered within an ecosystem and of benefit to humans	[26, 84]	
Exposure Characterisation	Quantitative estimation of the likely exposure of other biota and the environment to the transgenic, conducted subsequent to the problem formulation in an ERA	[26]	
Exposure Route	Possible route by which direct and indirect exposure to a potential harm may occur	[26]	
Fitness	Success of an individual in surviving and reproducing, measured by the individual‘s genetic contribution to the next generation and subsequent generations	[26]	
Harm	Adverse effect on something of value, relevant to an identified protection goal	[41]	
Hazard	Potential adverse effects that can lead harm to the environment or health	[26]	
Hazard Characterisation	Qualitative and/or quantitative evaluation of environmental or health adverse effects, conducted subsequent to the problem formulation in an ERA	[26]	
Indirect Effect	Effects on individual organisms in the wider environment without immediate contact with the transgenic	[26]	
Limits of Concern	Minimum ecological effects set for each assessment endpoint that are deemed both biologically relevant and of sufficient magnitude to cause harm.	[26]	
Measurement Endpoint	Measurable characteristic that is related to the environmental or health value chosen as the assessment endpoint	[26]	
Plausible Pathway to Potential Harm	Causal chain of events that would need to occur for a potential harm to a protection goal to be realised, often referred to a “pathway to harm” in the literature for brevity	[41]	
Potential Harm	Theoretical adverse outcome relevant to a protection goal	[41]	
Problem Formulation	First step in ERA where policy goals are identified, and pathways to harm, risk hypotheses and analysis plans are defining to guide the evaluation of data in the next steps of ERA.	[26, 37]	
Protection Goal	Policy and legislation defining environmental or health resources to be protected, the degree of protection they deserve, or the maximum impacts that should be tolerated.	[38]	
Risk	Combination of the magnitude of a hazard, if it occurs, and the likelihood that it occurs	[27]	
Risk Hypothesis	Hypothesis generated in problem formulation for specific step in pathway to potential harm such that no more harm or risk will occur to a protection goal than via existing activities	[41]	
Valued species	Species that is keystone, charismatic, threatened or endangered; identified and characterised from National Biodiversity Strategies and Actions Plans as set out by the CBD or the IUCN Red List of Threatened Species, and locally derived knowledge.	[81, 82]	
Vectorial capacity (V)	Total number of potentially infectious bites that would eventually arise from all the mosquitoes biting a single completely infectious (i.e., all mosquito bites result in infection) host on a single day, with individual elements that contribute to this value identified below:	[88–90]	

**Table 2:** List of identified plausible pathways to potential harm from field release of *dsxF*^*CRISPRh*^ transgenics in West Africa

Protection goal	Plausible pathway to potential Harm			Cause of potential harm		Effect of potential harm		Correlation of Exposure Levels with Transgene Efficacy		Relevance to ERAs for other transgenic mosquito strains
Biodiversity	1	Potential toxicological effects of *dsxF*^*CRISPRh*^ transgenics on NTOs could reduce ecosystem services.		Transgenic contains toxin or allergen		Direct: Reduced density of valued species or ecosystem services		Positive with gene drive; negative with population suppression; dependent on presence of transgene		All transgenic strains
	2	Potentially broader tolerances for humidity, temperature, salinity, or desiccation in *dsxF*^*CRISPRh*^ transgenics could reduce densities of valued species or ecosystem services.		Increased fitness in transgenic; changes in competitive interactions		Direct: Reduced density of valued species or ecosystem services		Positive with gene drive; negative with population suppression; dependent on presence of transgene		All transgenic strains
	3	Potentially cumulative Cas9/gRNA off-target or retargeted nuclease activity in *dsxF*^*CRISPRh*^ transgenics could cause broader tolerances for humidity, temperature, salinity, or egg desiccation to reduce densities of valued species or ecosystem services.		Off-target or re-targeted mutations; increased fitness in transgenic; changes in competitive interactions		Direct: Reduced density of valued species or ecosystem services		Positive with gene drive; negative with population suppression; independent of presence of transgene		All CRISPR-Cas9-based transgenic strains
	4	Potential horizontal gene flow of the *dsxF*^*CRISPRh*^ transgene that would contain construct backbone sequences could confer a growth advantage to bacteria that are pathogenic to a valued species, thus reducing densities of valued species or ecosystem services.		Gene flow to NTOs		Indirect: Reduced density of valued species or ecosystem services		Positive with gene drive; negative with population suppression; dependent on presence of transgene		All transgenic strains
	5	Potential horizontal gene flow of the *dsxF*^*CRISPRh*^ transgene to a NTO eukaryote could lead to its unintended population suppression, thus reducing densities of valued species or ecosystem services.		Gene flow to NTOs		Indirect: Reduced density of valued species or ecosystem services		Positive with gene drive; negative with population suppression; dependent on presence of transgene		All population suppression gene drive transgenic strains
	6	Reduction in densities of valued species or ecosystem services could be caused by their increased consumption by a predator.		Transgenic has altered physiology, anatomy, or behaviour; population suppression; changes in predator-prey interactions		Indirect: Reduced density of valued species or ecosystem services		Positive with gene drive; positive with population suppression; independent of presence of transgene in some circumstances		All population suppression gene drive transgenic strains, but potentially applicable any other successful gene drive transgenic strains
	7	Upon population suppression of *Anopheles gambiae* via gene drive, its niche could be occupied by competitor species that could cause suppression of a valued species to affect ecosystem services.		Changes in competitive interactions		Indirect: Reduced density of valued species or ecosystem services		Positive with gene drive; positive with population suppression; independent of presence of transgene		All population suppression gene drive transgenic strains, but potentially applicable any other successful vector control approaches
	8	Potential reductions in densities of valued species or ecosystem servicers due to poor nutrient composition of aquatic habitats could be caused by potentially increased *dsxF*^*CRISPRh*^ transgenic larval mortality.		Fitness costs in transgenic		Indirect: Reduced density of valued species or ecosystem services		Positive with gene drive; negative with population suppression; dependent on presence of transgene		Gene drive transgenic strains
Water quality	9	Potential adverse impact on quality of water, and its flora and fauna, from reduced nutrient composition of aquatic habitats could be caused by potential toxicity of *dsxF*^*CRISPRh*^ transgenic products.		Fitness costs in transgenic; transgenic contains toxin or allergen.		Indirect: Toxic water quality for NTOs		Positive with gene drive; negative with population suppression; dependent on presence of transgene		All transgenic strains
	10	Potential adverse impact on drinking water in aquatic habitats could be caused by potentially higher mortality of *dsxF*^*CRISPRh*^ transgenic larvae.		Fitness costs in transgenic		Indirect: Reduced water quality for humans and livestock		Positive with gene drive; negative with population suppression; dependent on presence of transgene		All transgenic strains
Human health	11	Transgenic proteins could cause specific allergic or toxicological responses in humans from *dsxF*^*CRISPRh*^ transgenic bites beyond responses to non-transgenic bites.		Transgenic contains toxin or allergen		Direct: Increased allergic or immune responses in humans; increased toxicity in humans		Independent of efficacy of gene drive or population suppression as defined by allergic responses in individual humans; dependent on presence of transgene		All transgenic strains
	12	Potential incidental ingestion or inhalation of *dsxF*^*CRISPRh*^ transgenic material could cause specific allergic responses in humans beyond responses to non-transgenic material.		Transgenic contains toxin or allergen		Direct: Increased allergic or immune responses in humans		Independent of efficacy of gene drive or population suppression as defined by allergic responses in individual humans; dependent on presence of transgene		All transgenic strains
	13	Increased allergenicity in humans could occur from potentially altered levels of endogenous allergens in *dsxF*^*CRISPRh*^ transgenics.		Transgenic contains toxin or allergen		Direct: Increased allergic or immune responses in humans		Independent of efficacy of gene drive or population suppression as defined by allergic responses in individual humans; dependent on presence of transgene		All transgenic strains
	14	Potentially decreased mosquito defence response to pathogen in *dsxF*^*CRISPRh*^ transgenics from altered levels of endogenous RNA, protein or microbiome could lead to increased human disease.		Transgenic has altered physiology, anatomy, or behaviour; increased vector competence in transgenic		Direct: Increased disease transmission in humans		Positive with gene drive; negative with population suppression; dependent on presence of transgene		Arises from specific anatomical alterations in homozygous *dsxF*^*CRISPRh*^ transgenics but could be applicable to other transgenic strains
	15	Potentially decreased human defence response to pathogen from altered levels of endogenous RNA or protein in the saliva *dsxF*^*CRISPRh*^ transgenics could lead to increased disease in humans.		Transgenic has altered physiology, anatomy, or behaviour		Direct: Increased disease transmission in humans		Positive with gene drive; negative with population suppression; dependent on presence of transgene		Arises from specific anatomical alterations in homozygous *dsxF*^*CRISPRh*^ transgenics but could be applicable to other transgenic strains
	16	Potential immunopathological responses via biting exposure to gRNA expressed in saliva of *dsxF*^*CRISPRh*^ transgenic could lead to increases in morbidity and mortality in humans.		Transgenic has altered physiology, anatomy, or behaviour		Direct: Increased allergic or immune responses in humans		Independent of efficacy of gene drive or population suppression as defined by allergic responses in individual humans; dependent on presence of transgene		All CRISPR-Cas9-based transgenic strains
	17	Potential secondary toxicological effects in humans from consuming NTOs which would have fed on *dsxF*^*CRISPRh*^ transgenics.		Transgenic contains toxin or allergen		Indirect: Increased toxicity in humans		Positive with gene drive; negative with population suppression; dependent on presence of transgene		All transgenic strains
	18	Potentially increased fitness, including insecticide resistance, of *dsxF*^*CRISPRh*^ transgenics could increase disease transmission in humans.		Transgenic has altered physiology, anatomy, or behaviour; increased fitness in transgenic		Direct: Increased disease transmission in humans		Positive with gene drive; negative with population suppression; dependent on presence of transgene		All transgenic strains
	19	Potentially increased biting rate of *dsxF*^*CRISPRh*^ transgenics could increase disease transmission in humans.		Transgenic has altered physiology, anatomy, or behaviour; increased biting rates		Direct: Increased disease transmission in humans		Positive with gene drive; negative with population suppression; dependent on presence of transgene		Arises from specific anatomical alterations in homozygous *dsxF*^*CRISPRh*^ transgenics but could be applicable to other transgenic strains
	20	Potentially increased vector competence in *dsxF*^*CRISPRh*^ transgenics could increase disease transmission in humans.		Transgenic has altered physiology, anatomy, or behaviour; increased vector competence in transgenic		Direct: Increased disease transmission in humans		Positive with gene drive; negative with population suppression; dependent on presence of transgene		All transgenic strains
	21	Potentially altered anatomy, or host-seeking behaviour, in *dsxF*^*CRISPRh*^ transgenics could increase the transmission of human diseases, including lymphatic filariasis.		Transgenic has altered physiology, anatomy, or behaviour; increased vector competence in transgenic; increased biting rates		Direct: Increased disease transmission in humans		Positive with gene drive; negative with population suppression; dependent on presence of transgene		Arises from specific anatomical alterations in homozygous *dsxF*^*CRISPRh*^ transgenics but could be applicable to other transgenic strains
	22	Potentially altered anatomy in *dsxF*^*CRISPRh*^ transgenics could lead them to vector human disease not previously-vectored by *Anopheles gambiae*.		Transgenic has altered physiology, anatomy, or behaviour; increased vector competence in transgenic		Direct: Novel disease transmission in humans		Positive with gene drive; negative with population suppression; dependent on presence of transgene		Arises from specific anatomical alterations in homozygous *dsxF*^*CRISPRh*^ transgenics but could be applicable to other transgenic strains
	23	Potentially altered physiology in *dsxF*^*CRISPRh*^ transgenics could increase disease transmission in humans.		Transgenic has altered physiology, anatomy, or behaviour; increased vector competence in transgenic		Direct: Increased disease transmission in humans		Positive with gene drive; negative with population suppression; dependent on presence of transgene		Arises from specific anatomical alterations in homozygous *dsxF*^*CRISPRh*^ transgenics but could be applicable to other transgenic strains
	24	Potentially altered physiology in *dsxF*^*CRISPRh*^ transgenic could lead them to vector human disease not previously-vectored by *Anopheles gambiae.*		Transgenic has altered physiology, anatomy, or behaviour; increased vector competence in transgenic		Direct: Novel disease transmission in humans		Positive with gene drive; negative with population suppression; dependent on presence of transgene		Arises from specific anatomical alterations in homozygous *dsxF*^*CRISPRh*^ transgenics but could be applicable to other transgenic strains
	25	Potentially cumulative Cas9/gRNA off-target or retargeted nuclease activity in *dsxF*^*CRISPRh*^ transgenics could cause heritable increase in insecticide resistance, fitness or vector competence to increase human disease.		Off-target or re-targeted mutations; transgenic has altered physiology, anatomy, or behaviour; increased vector competence in transgenic; increased fitness in transgenic		Direct: Increased disease transmission in humans		Positive with gene drive; negative with population suppression; independent of presence of transgene		All CRISPR-Cas9-based transgenic strains
	26	Potentially broader tolerances for humidity, temperature, salinity, or desiccation in *dsxF*^*CRISPRh*^ transgenic could lead to increased disease transmission in humans.		Transgenic has altered physiology, anatomy, or behaviour; increased transgenic fitness		Direct: Increased disease transmission in humans		Positive with gene drive; negative with population suppression; dependent on presence of transgene		All transgenic strains
	27	Increased or novel human disease transmission could be caused by replacement of *Anopheles gambiae* niche with another disease vector.		Population suppression; changes in competitive interactions		Indirect: Increased disease transmission in humans		Positive with gene drive; positive with population suppression; independent of presence of transgene		All population suppression gene drive transgenic strains, but potentially applicable to any other successful vector control approaches
	28	Potential toxicological effects of *dsxF*^*CRISPRh*^ transgenics on NTOs could increase disease transmission in humans.		Transgenic contains toxin or allergen		Indirect: increased disease transmission in humans		Positive with gene drive; negative with population suppression; dependent on presence of transgene		All transgenic strains
	29	Potentially reduced density of a predator species caused by population suppression of *Anopheles gambiae* could lead to increases in density of another human disease vector species.		Population suppression; changes in predator-prey interactions		Indirect: increased disease transmission in humans		Positive with gene drive; positive with population suppression; independent of presence of transgene		All population suppression gene drive transgenic strains, but potentially applicable to any other successful vector control approaches
	30	Potential increases in disease levels beyond those pre-gene drive intervention following a resurgence in pathogen transmission after initial population suppression would have reduced human immunity to pathogen.		Population suppression; changes in herd immunity		Indirect: Increased disease transmission in humans		Positive with gene drive; positive with population suppression; independent of presence of transgene		All population suppression gene drive transgenic strains, but potentially applicable to any other successful vector control approaches
Animal health	31	Potential toxicity in livestock from *dsxF*^*CRISPRh*^ transgenic proteins in saliva.		Transgenic contains toxin or allergen		Direct: Increased toxicity in livestock		Positive with gene drive; negative with population suppression; dependent on presence of transgene		All transgenic strains
	32	Potentially decreased mosquito defence response to pathogen in *dsxF*^*CRISPRh*^ transgenics from altered levels of endogenous RNA, protein or microbiome could lead to increased disease in livestock.		Transgenic has altered physiology, anatomy, or behaviour; increased vector competence in transgenic		Direct: Increased disease transmission in livestock		Positive with gene drive; negative with population suppression; dependent on presence of transgene		Arises from specific anatomical alterations in homozygous *dsxF*^*CRISPRh*^ transgenics but could be applicable to other transgenic strains
	33	Potentially decreased livestock defence response to pathogen from altered levels of endogenous RNA or protein in saliva of *dsxF*^*CRISPRh*^ transgenics could lead to increased disease in livestock.		Transgenic has altered physiology, anatomy, or behaviour		Direct: Increased disease transmission in livestock		Positive with gene drive; negative with population suppression; dependent on presence of transgene		Arises from specific anatomical alterations in homozygous *dsxF*^*CRISPRh*^ transgenics but could be applicable to other transgenic strains
	34	Potentially increased fitness, including insecticide resistance, of *dsxF*^*CRISPRh*^ transgenic could increase disease transmission in livestock.		Transgenic has altered physiology, anatomy, or behaviour; increased fitness in transgenic		Direct: Increased disease transmission in livestock		Positive with gene drive; negative with population suppression; dependent on presence of transgene		All transgenic strains
	35	Potentially increased biting rate of *dsxF*^*CRISPRh*^ transgenic could increase disease transmission in livestock.		Transgenic has altered physiology, anatomy, or behaviour; increased biting rates		Direct: Increased disease transmission in livestock		Positive with gene drive; negative with population suppression; dependent on presence of transgene		Arises from specific anatomical alterations in homozygous *dsxF*^*CRISPRh*^ transgenics but could be applicable to other transgenic strains
	36	Potentially increased vector competence of *dsxF*^*CRISPRh*^ transgenic could increase disease transmission in livestock.		Transgenic has altered physiology, anatomy, or behaviour; increased vector competence in transgenic		Direct: Increased disease transmission in livestock		Positive with gene drive; negative with population suppression; dependent on presence of transgene		All transgenic strains
	37	Potentially altered anatomy, or host-seeking behaviour, in *dsxF*^*CRISPRh*^ transgenic could increase disease transmission in livestock.		Transgenic has altered physiology, anatomy, or behaviour; increased vector competence in transgenic; increased biting rates		Direct: Increased disease transmission in livestock		Positive with gene drive; negative with population suppression; dependent on presence of transgene		Arises from specific anatomical alterations in homozygous *dsxF*^*CRISPRh*^ transgenics but could be applicable to other transgenic strains
	38	Potentially altered anatomy in *dsxF*^*CRISPRh*^ transgenic could lead it to vector livestock animal disease not previously-vectored by *Anopheles gambiae*.		Transgenic has altered physiology, anatomy, or behaviour; increased vector competence in transgenic; increased biting rates		Direct: Novel disease transmission in livestock		Positive with gene drive; negative with population suppression; dependent on presence of transgene		Arises from specific anatomical alterations in homozygous *dsxF*^*CRISPRh*^ transgenics but could be applicable to other transgenic strains
	39	Potentially altered physiology in *dsxF*^*CRISPRh*^ transgenic could increase disease transmission in livestock.		Transgenic has altered physiology, anatomy, or behaviour; increased vector competence in transgenic		Direct: Increased disease transmission in livestock		Positive with gene drive; negative with population suppression; dependent on presence of transgene		Arises from specific anatomical alterations in homozygous *dsxF*^*CRISPRh*^ transgenics but could be applicable to other transgenic strains
	40	Potentially altered physiology in *dsxF*^*CRISPRh*^ transgenic could lead it to vector animal disease not previously-vectored by *Anopheles gambiae*.		Transgenic has altered physiology, anatomy, or behaviour; increased vector competence in transgenic		Direct: Novel disease transmission in livestock		Positive with gene drive; negative with population suppression; dependent on presence of transgene		Arises from specific anatomical alterations in homozygous *dsxF*^*CRISPRh*^ transgenics but could be applicable to other transgenic strains
	41	Potentially cumulative Cas9/gRNA off-target or retargeted nuclease activity in *dsxF*^*CRISPRh*^ transgenic could cause increase in insecticide resistance, fitness or vector competence to increase disease transmission in livestock.		Off-target or re-targeted mutations; transgenic has altered physiology, anatomy, or behaviour; increased fitness in transgenic; increased vector competence in transgenic		Direct: Increased disease transmission in livestock		Positive with gene drive; negative with population suppression; independent of presence of transgene		All CRISPR-Cas9-based transgenic strains
	42	Potentially broader tolerances for humidity, temperature, salinity, or desiccation in *dsxF*^*CRISPRh*^ transgenics could lead to increased disease transmission in livestock.		Transgenic has altered physiology, anatomy, or behaviour; increased fitness in transgenic		Direct: Increased disease transmission in livestock		Positive with gene drive; negative with population suppression; dependent on presence of transgene		All transgenic strains
	43	Increased or novel disease transmission in livestock animals could be caused by replacement of *Anopheles gambiae* niche with another disease vector.		Population suppression: changes in competitive interactions		Indirect: Increased disease transmission in livestock		Positive with gene drive; positive with population suppression; independent of presence of transgene		All population suppression gene drive transgenic strains, but potentially applicable to any other successful vector control approaches
	44	Potential toxicological effects of *dsxF*^*CRISPRh*^ transgenics on NTOs could increase disease transmission in livestock.		Transgenic contains toxin or allergen		Indirect: Increased disease transmission in livestock		Positive with gene drive; negative with population suppression; dependent on presence of transgene		All transgenic strains
	45	Reduced density of a predator species that could be caused by population suppression of *Anopheles gambiae* could lead to increases in density of another animal disease vector species.		Population suppression: changes in predator-prey interactions		Indirect: Increased disease transmission in livestock		Positive with gene drive; positive with population suppression; independent of presence of transgene		All population suppression gene drive transgenic strains, but potentially applicable to any other successful vector control approaches
	46	Potential increases in livestock disease beyond pre-gene drive intervention levels following resurgence in pathogen transmission after initial population suppression would have reduced livestock immunity to pathogen.		Population suppression: changes in herd immunity		Indirect: Increased disease transmission in livestock		Positive with gene drive; positive with population suppression; independent of presence of transgene		All population suppression gene drive transgenic strains, but potentially applicable to any other successful vector control approaches

Problem formulation has previously been used to identify potential harms or hazards and routes of exposure for the control of insect pests [43] and potential releases of generic gene drive systems for malaria vector control in Africa [44–46]. Romeis et al. [43] reported on pathways to harm for population suppression gene drive in the agricultural pest *Drosophila suzukii*, identifying (i) indirect food-web effects; (ii) toxicity from the transgene; and, (iii) gene transfer to other species as potential harms. David et al. [44] identified ecological harms as a first step towards an ERA, distinguishing between transient and steady state harms but without specific reference to protection goals or pathways. Roberts et al. [45] derived protection goals on human health, animal health, biodiversity and water quality for broad classes of gene drive, identifying numerous high-level potential harms belonging to each of these categories, although they were not mapped onto individual pathways to harm, so that risk hypotheses and analysis plans were not developed or reported. Based on a series of four workshops in different African countries, Teem et al. [46] reported a problem formulation exercise based on four different types of gene drive to control malaria, identifying many protection goals similar to those previously reported [45], along with six high-level, consensus pathways to harm. However, the authors recognized that the approach was not designed to constitute a comprehensive first step in a specific ERA exercise and developed only limited analysis plans for each pathway [46].

Here, problem formulation was used to systematically and comprehensively map 46 plausible pathways to potential harm from the simulated investigational release in West Africa of a population suppression gene drive that would employ the CRISPR-Cas9 system to target the *doublesex* female fertility gene (*dsxF*^*CRISPRh*^) [17, 19]. For each of these pathways, risk hypotheses and an analysis plan were developed [47], which will be used to inform subsequent steps of hazard and exposure characterization in an ERA for population suppression gene drive in West Africa.

## Methods

### Defining ***Anopheles gambiae***

The *Anopheles gambiae* species complex, or *Anopheles gambiae senso lato* (*s.l.*), is currently considered to be made up of nine cryptic species, namely *Anopheles amharicus*, also referred to in earlier publications as “species B of *Anopheles quadriannulatus*” [48]; *Anopheles arabiensis*, referred to in earlier publications as “species B of *An. gambiae*” [49]; *Anopheles bwambae*, referred to in earlier publications as “species D of *An. gambiae*” [49]; *Anopheles coluzzii*, referred to as “the M form of *An. gambiae sensu stricto* (*s.s.*)” in earlier publications [49]; *Anopheles fontenillei*; *An. gambiae s.s.*, referred to as “the S form of *An. gambiae s.s*.” in earlier publications [49]; *Anopheles melas*; *Anopheles merus;* and *An. quadriannulatus*, referred to in earlier publications as “species C of *An. gambiae*” or “species A of *An. quadriannulatus*” [48–52].

Throughout this paper, the term *An. gambiae* is used when referring to the *An. gambiae* species complex and *An. gambiae s.s.* when referring to that particular species within the *An. gambiae* complex. *Anopheles gambiae s.s.* and *An. coluzzii* were previously considered to be a single species, known as “species A of *An. gambiae*” in earlier publications, but appear to have been undergoing a process of speciation [49]. Of species from the *An. gambiae* complex, *An. coluzzii*, *An. gambiae s.s.* and *An. arabiensis* have been identified as dominant malaria vectors [53], while others are considered to be minor vectors of malaria, often because of their preference for blood meals from animal hosts [54–56].

### Defining the transgenic strain and release conditions

This problem formulation was based on a theoretical release of a strain of *An. coluzzii*, which contains a transgene encoding the fluorescent marker gene, *DsRed*, and the CRISPR-Cas9 system to target conserved sequences of a female-specific isoform of the haplo-sufficient *doublesex* gene (*dsxF*) [17]. This transgene (*dsxF*^*CRISPRh*^) disrupts the female-specific exon of the *doublesex* gene so that homozygous transgenic females (*dsxF*^*CRISPRh*^*/dsxF*^*CRISPRh*^) are completely infertile, have altered morphology and do not bite, and therefore neither feed on blood nor transmit malaria. By contrast, heterozygotes (*dsxF*^*CRISPRh*^/+) are typically fertile and can bite, although somatic mosaicism has been reported in some heterozygous females that had received the *dsxF*^*CRISPRh*^ allele paternally leading to anatomical alterations of varying penetrance [17]. The hypothetical field protocol follows a scenario modelled in the simulated annual releases of 5000 transgenic heterozygous males over a six-year period in 1% of human settlements in a 1 million sq. km grid in West Africa, as described in North et al. [19].

Over this area, the predominant malaria vectors are the species *An. gambiae s.s*. and *An. coluzzii* [57–59]. *Anopheles gambiae s.s.* favours laying eggs in aquatic habitats that are small, clean, sunlit, lacking vegetation and ephemeral, so that they quickly dry out and thus development of aquatic stages of this species is relatively fast. By contrast, *An. coluzzii* favours larger, more permanent bodies of water, often with overhanging vegetation, such as the banks of rivers with slow-moving water or irrigation channels of rice paddies, where larval developmental times typically are longer [57–59].

The region of release consists of both savannah and semi-arid regions in the Sahel with areas experiencing a range of seasonality: some areas contain year-round larval aquatic habitats, whilst others have two main seasons, a rainy or wet season which lasts roughly from April to October and a dry season, which thus affects the availability of most aquatic habitats of *An. gambiae.* In addition, human settlements in the area also vary significantly in their degree of connectedness with other settlements, thus affecting the ease with which vectors can migrate from one human population to another.

**Fig. 1:**
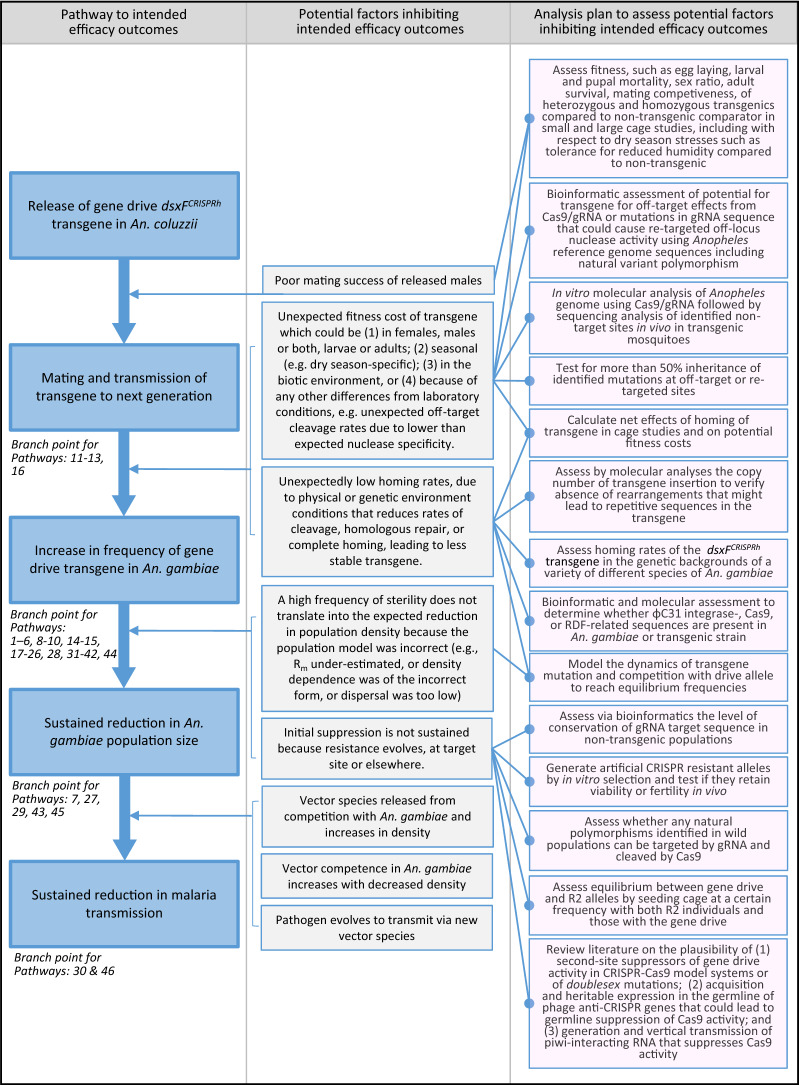
Pathway to intended efficacy outcomes from *dsxF*^*CRISPRh*^ transgenic releases. Potential factors that could inhibit steps in the efficacy pathway, the point at which they could occur, analysis plan to detect the presence of such factors and branch points from intended efficacy outcomes for individual potential harms. For each pathway to harm in this study, the first stages of the pathway involving intended efficacy outcomes are illustrated in dark blue, with subsequent steps in the pathway specific to the occurrence of that harm are shown in lighter blue. Third to last potential factor inhibiting intended efficacy outcomes is addressed via Pathways 7, 27, 29, 43 and 45 (see Figs. 9, 29, 45 and 47, respectively). Second last potential factor inhibiting intended efficacy outcomes would be assessed in analysis plan of Pathway 20 via modelling. Last potential factor inhibiting intended efficacy outcomes could occur in the absence of the intervention and thus “*will do no more harm to human health than wild-type mosquitoes*” [30]. Analysis plan represents data gathering pre-release only. Further assessments and field monitoring would accompany field releases but be addressed in subsequent risk management stages of the ERA

A broad outline of intended efficacy outcomes from release of this strain is illustrated in Fig. 1. Spatial modelling has indicated that releases in this region of relatively small numbers of *dsxF*^*CRISPRh*^ transgenics are likely to lead to a range of entomological outcomes from near-complete population suppression to intermediate states where transgenic and wild-type alleles reach equilibrium levels within the population depending on the seasonality and connectivity of the release location to other human settlements [19, 60].

### Defining the population dynamics of gene drive genotypic risk profiles

**Fig. 2:**
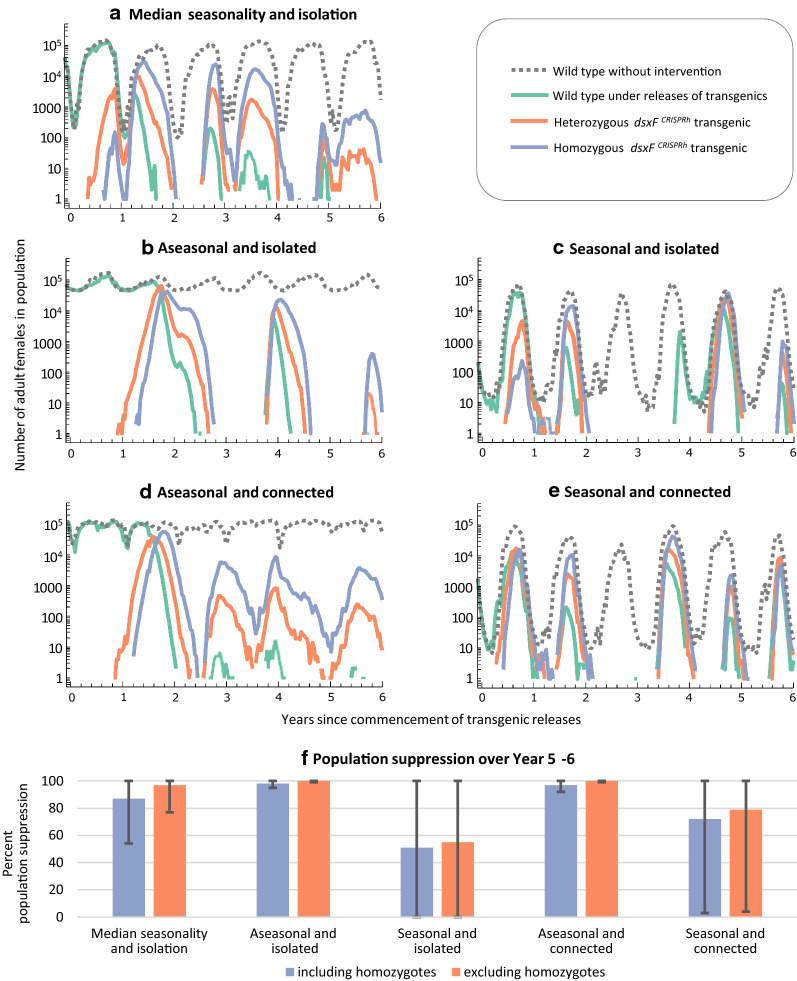
Spatial and temporal heterogeneity in genotypic dynamics and population suppression following simulated releases of *dsxF*^*CRISPRh*^ transgenics. A previously reported model [19] was used to create plots showing typical dynamics of transgenic heterozygotes (*dsxF*^*CRISPRh*^/+) and homozygotes (*dsxF*^*CRISPRh*^/*dsxF*^*CRISPRh*^) at five sites (villages) that differ in their extent of seasonality and isolation from other human settlements. Hypothetical field protocol for problem formulation follows scenario modelled in simulated annual releases of 5000 transgenic heterozygous males over a 6-year period in 1% of human settlements (n = 434 of 42,260) in a 1 million sq. km grid in West Africa [19]. Representative simulations of genotypic and population dynamics are shown on a logarithmic scale for adult female population size at five site conditions: **a** Median seasonality and isolation: both seasonality and isolation from other human settlements in the 50th percentile; **b** Aseasonal and isolated: both aseasonality and isolation from other human settlements in the 95th percentile; **c** Seasonal and isolated: both seasonality and isolation from other human settlements in the 95th percentile; **d** Aseasonal and connected: both aseasonality and connectedness to other human settlements in the 95th percentile; **e** Seasonal and connected: both seasonality and connectedness to other human settlements in the 95th percentile. Solid lines in line graphs show numbers of adult female genotypes in populations where transgenic releases are occurring. Dotted lines represent simulations of numbers of wild type females in populations in the absence of population suppression gene drive. The low number (< 3%) [19] of non-functional cleavage resistant alleles [140] have been excluded here to provide visual clarity to the graphs. **f** Population suppression from gene drive intervention over calendar Year 5–6 compared to the year before transgenic releases. Means were obtained from ten simulations each of the different site conditions, controlled for rainfall, with error bars indicating minimum and maximum values observed

The dynamics of the increase in frequency of the transgene in mosquito populations will vary across the landscapes where releases might take place, due to spatial and temporal heterogeneity in environmental conditions. In particular, differing levels of seasonality from one location to another may lead to spatial variation in risk exposure [19]. Using the model developed by North et al. [19], a specific analysis was carried out to differentiate between the frequency of transgenic heterozygotes (*dsxF*^*CRISPRh*^/+) and homozygotes (*dsxF*^*CRISPRh*^*/dsxF*^*CRISPRh*^) under different conditions of seasonality and connectedness between human settlements (Fig. 2). Simulations found that the transgene typically establishes in local (village) populations via heterozygous mosquitoes, which then increase in abundance to precipitate the production of homozygotes. In aseasonal populations, the number of homozygotes will eventually surpass that of heterozygotes and this dynamic results in sustained population suppression. In populations that have strong seasonal fluctuations in size, however, both the transgenic genotypes may establish concurrently before being maintained at roughly equivalent levels in yearly cycles, resulting in more limited population suppression (Fig. 2). Seasonality may therefore influence risk exposure both through its effect on population suppression *per se*, and by how it mediates the relative frequencies of biting heterozygotes and non-biting homozygotes.

### Defining target organisms

Species of the *An. gambiae* complex show only partial reproductive isolation and, for at least some of the species that live in sympatry, hybrids have been observed in nature at low frequencies [51, 61–66], and gene flow has been inferred from genomic analyses [65–70]. Moreover, because the guide RNA target sequence of the *dsxF*^*CRISPRh*^ transgene is conserved in all of the above species examined [17], the transfer of this transgene between any of these species via hybridization may lead to functional gene drive and population suppression in those species. It is, however, possible that some species of the complex would undergo less efficacious population suppression than others, should they be inefficiently targeted by gene drive via assortative mating, or where there might be species-specific resistance (Fig. 1).

The most closely related species to those within the *An. gambiae* complex is *Anopheles christyi*, which is also in the pyretophorus series of sub-genus *Cellia* [71], but differs morphologically and is genetically distinct from species of *An. gambiae*, being separated by circa 9 million years of evolution [72]. The absence of observed gene flow between species of *An. gambiae* and *An. christyi* supports the lack of any significant hybridization between these species so that, for even less closely related species of *Anopheles*, hybridization is considered implausible. Moreover, in species of *Anopheles* more distantly related to *An. gambiae* than *An. christyi*, the guide RNA target DNA sequence of the *dsxF*^*CRISPRh*^ transgene diverges from that found in *An. gambiae* [17].

Therefore, for the purposes of this problem formulation, the nine species of *An. gambiae* listed above were considered to be ‘inside the complex’ and, therefore, TOs of the population suppression gene drive. All other species that are ‘outside the complex’ were therefore regarded as non-target organisms (NTOs), including the remaining 316 mosquito species (Family: Culicidae) that have been reported in countries of West Africa lying within the 1 million sq. km grid of simulated field releases from this study, namely Burkina Faso, Mali, Côte D’Ivoire, Ghana, Togo, Bénin, Nigeria, and Niger [19, 60, 73, 74].

### Defining intended efficacy outcomes and their relationships to potential harms

The high-level pathway to intended efficacy outcomes was established and potential factors that could inhibit steps in this ‘efficacy pathway’ and the point at which they could occur were defined (Fig. 1), and from this an analysis plan was developed. Certain factors that could lead to a loss of efficacy are the subject of active screening during standard strain development processes before field release: for example, unexpected copy number or re-arrangements of the transgene would be detected by sequencing, or significant fitness costs from the transgene should be observed in insectary studies. Moreover, further assessments and field monitoring would accompany field releases, the specifications for which would be determined in subsequent risk management stages of an ERA.

While loss of efficacy following an investigational release of a population suppression gene drive would not be a harm *per se*, it would likely alter exposure levels to potential harms arising from both wild-type and *dsxF*^*CRISPRh*^ transgenic vectors. For each pathway, the first steps involving intended efficacy outcomes, as illustrated in Fig. 1, are shown in dark blue, with branch points to subsequent steps in the pathway specific to the occurrence of that potential harm shown in lighter blue.

### Defining protection goals and plausible pathways to potential harm

The protection goals used in this problem formulation were informed by the outputs of four, 4-day workshops organized by New Partnership for Africa’s Development of the African Union Development Agency (AUDA-NEPAD) at Accra, Ghana; Nairobi, Kenya; Gaborone, Botswana; and, Libreville, Gabon between 2016 and 2018 [46] and a 2-day workshop organized by the Foundation for the National Institutes of Health (FNIH) at Accra, Ghana in February 2019. A 3-day workshop organized by the FNIH at Reston, USA in 2016 [45], extensive literature assessment, and ongoing dialogue with numerous scientific, risk and regulatory experts also contributed to this exercise. Four broad protection goals (biodiversity, water quality, human health, animal health) were identified from these previously published problem formulation exercises [45, 46]. In contrast to Teem et al. [46], but in keeping with Roberts et al. [45], soil quality was discounted as a broad protection goal that could plausibly be affected by population suppression gene drive, given that *An. gambiae* reflects a relatively insignificant proportion of the terrestrial biomass [75].

Importantly, ERA does not formally include socio-economic or legal issues, such as the potential for transboundary movement of transgenics, despite these warranting further exploration in the context of gene drive organisms that are anticipated to cross national borders. Additionally, some pathways which could extend to further potential economic and social harms beyond the direct biosafety considerations here, such as those involving potential harms to livestock, were considered out of scope for this problem formulation. Instead, such issues could be separately addressed under assessment frameworks explicitly suited to those endpoints, such as social, economic and health impact assessment [76, 77], as well as via any regional harmonization of regulatory processes [78, 79].

Potential harms to protection goals, their pathways and their analysis plans were refined in an iterative fashion involving multiple working group meetings amongst the authors, as well as subsequent review of conceptual models by 14 experts in medical entomology, vector biology, ecology, population genetics, molecular biology, protein engineering, and modelling from Africa, Europe and North America (see Acknowledgements). The plausibility of each pathway was considered, based on known biological and environmental evidence, and literature and the causal chain of events, or plausible pathway, which would be required for each potential harm to occur, were mapped out in a logical, linear order.

As part of the systematic process to validate pathways in the ERA, risk hypotheses for critical steps in each pathway were developed [41] and measurement endpoints were next defined to corroborate or invalidate a given risk hypothesis. By convention, each step of every pathway should ideally be accompanied by a corresponding risk hypothesis and measurement endpoints that could unambiguously test the veracity of each of those hypotheses [41]. However, only key risk hypotheses and measurement endpoints were developed for each of the pathways here, principally to avoid duplication and to provide a transparent focus on what was considered to be the most salient elements contributing to potential harms. Additionally, other potential sources of evidence were identified that could act to reduce areas of uncertainty in certain plausible pathways. Together, measurement endpoints and potential evidence to reduce uncertainty constituted the analysis plan for each pathway. Analysis plans will therefore form a crucial aspect of the evidence base supporting subsequent stages of an ERA for the release of this population suppression gene drive in West Africa. Where relevant, an iterative and flexible tiered approach will be applied to analysis plans in both the testing of measurement endpoints and sourcing of other potential evidence in order to carry out a process that is relevant to further decisions, demonstrates confidence in safe development and is efficient in providing evidence for design and assessment [47, 80].

### Defining ‘fitness’

The term ‘fitness’ is used in many of the pathways to potential harm (Table 2). This is a deliberately broad, generic term that encompasses *viability*, such as larval and pupal survival, *fecundity*, such as number of eggs laid, or egg hatching rate, *fertility*, such as mating competitiveness, and *vigour*, such as adult longevity and survival under a range of environmental conditions, such as in the presence of insecticide, or over a range of temperatures, decreased humidity, or increased salinity. When appropriate and relevant, specific components of fitness were highlighted in individual pathways.

### Defining ‘valued species’ and ‘ecosystem services’

Many biodiversity protection goals are based on ‘valued species’ and ‘ecosystem services’. Valued species is a generic term that refers to any species that is i) keystone; ii) charismatic; iii) threatened; or, iv) endangered. Individual valued species will be identified and characterized in subsequent steps of an ERA using evidence from National Biodiversity Strategies and Action Plans as set out by the Convention on Biological Diversity [81], or the International Union for Conservation of Nature Red List of Threatened Species [82], augmented where appropriate by local knowledge and expertise of research colleagues from African partners in Target Malaria [83], as well as any socio-economic scoping or impact assessment studies which may be undertaken. More broadly, operational protection goals will be defined in subsequent stages of an ERA using a framework building on the concept of ecosystem services that can include (i) provisioning services such as food, water or energy; (ii) regulating services such as pollination, pest control or water purification; and, iii) supporting services such as oxygen generation or decomposition of organic matter and nutrient recycling [84–86]. This will be used to further refine assessment endpoints and their associated measurement endpoints, as well as limits of concern [26], in subsequent stages of an ERA for this population suppression gene drive [87].

### Defining ‘vectorial capacity’

‘Vectorial capacity’ is defined as the total number of potentially infectious bites that would eventually arise from all the mosquitoes biting a single completely infectious (i.e., all mosquito bites result in infection) host, be it human or animal, on a single day (Table 1) [88–90]. This value is relevant to a range of potential harms from the release of transgenic mosquitoes. The individual parameters contributing to vectorial capacity are shown in the equation at the bottom of Table 1 and include (i) host biting rate; (ii) vector competence; (iii) probability that a newly infected mosquito survives to become infectious; (iv) number of adult female mosquitoes per host; and, (v) daily probability that an infected adult female mosquito dies. Thus, changes to any one of these parameters in the *dsxF*^*CRISPRh*^ transgenic could alter the vectorial capacity for transmission of a given disease. Although the numerical value of this equation would be of limited use to vector control programmes, the equation does represent a useful tool to identify specific elements that contribute to disease transmission by vectors and can facilitate evaluation of the contribution that each of those elements make to transmission in modelling studies. In any pathway involving vector-borne human or animal disease, relevant measurement endpoints contributing to vectorial capacity are highlighted using the equation in Table 1.

### Defining off‐targeting and retargeting effects from the CRISPR/Cas9 system

Off-target mutations could theoretically occur in *dsxF*^*CRISPRh*^ transgenics by promiscuous cleavage by Cas9 at non-target genomic sequences followed by their misrepair [91]. Retargeting could also theoretically occur should the transgene mutate to modify the specificity of target sequence recognition and cleavage by Cas9, with misrepair leading to retargeted, off-locus mutations [92]. Off-target, or retargeted, mutations could then accumulate in target populations if they were to occur at sufficient frequency or be positively selected. They would not, in themselves, represent a harm unless they produce phenotypes that augment vectorial capacity or negatively impact on environmental protection goals.

The propensity for off-target effects is dependent on the uniqueness of the guide RNA target site; the efficiency, intracellular concentration and duration of the nuclease activity; and, the chromatin state of the tissues and cells in which the nuclease is expressed [91]. *In vitro* and *in vivo* molecular analyses have been developed to screen CRISPR-Cas9 transgenic lines for off-target mutations [93]. Bioinformatic software for the cogent design of guide RNAs [94] and promoters with optimal the intracellular concentration, duration and germline cell-specificity of Cas9 expression for on-target efficacy can be exploited to minimize the potential for off-target effects [91].

## Results

In total, for investigational field releases of the *dsxF*^*CRISPRh*^ transgene in West Africa, eight broad, potentially harmful effects were identified. These were stratified into 46 plausible pathways leading to potential harms to the four protection goals (Table 2). All 46 pathways are illustrated as Figs. 3, 4, 5, 6, 7, 8, 9, 10, 11, 12, 13, 14, 15, 16, 17, 18, 19, 20, 21, 22, 23, 24, 25, 26, 27, 28, 29, 30, 31, 32, 33, 34, 35, 36, 37, 38, 39, 40, 41, 42, 43, 44, 45, 46, 47, and 48, but 10 specific examples of conceptual models are described in the following four sections on protection goals, which in each case describe (i) plausible pathways to potential harm; (ii) risk hypotheses to interrogate individual steps in those pathways; and, (iii) analysis plans to corroborate or invalidate those hypotheses.

**Fig. 3:**
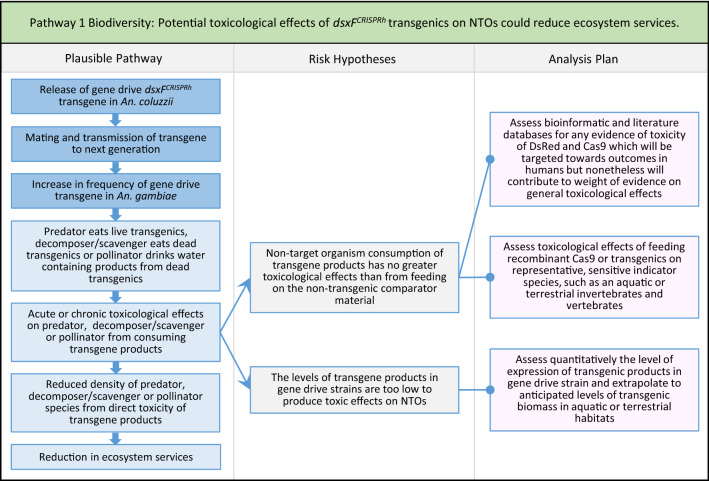
Pathway 1 Biodiversity: Potential toxicological effects of *dsxF*^*CRISPRh*^ transgenics on NTOs could reduce ecosystem services Where there would be effective population suppression, there would be reduced densities of *An. gambiae* and therefore less exposure of NTOs to any potential toxicological effects. In the analysis plan, bioinformatic and literature evidence of any toxicity of DsRed and Cas9 will likely be more targeted towards outcomes in humans but nonetheless could contribute to weight of evidence corroborating or falsifying the associated risk hypothesis. Defining the experimental conditions and choices of indicator species for toxicological studies will most likely involve discussion with national regulators, with reference to international regulatory guidance and best practice [26, 95]. For all potential harms, a tiered approach can be applied to the analysis plans in both the testing of measurement endpoints and sourcing of other potential evidence in order to ensure that identified studies are only conducted when they contribute directly to reductions in uncertainty in the ERA, thus preventing unnecessary and uninformative investigations [47, 80]. This plausible pathway to potential harm could also be relevant to water quality, human health and animal health protection goals, for example by increases in the densities of other pest or vector species if the predator were to feed on both *An. gambiae* and those other species

**Fig. 4:**
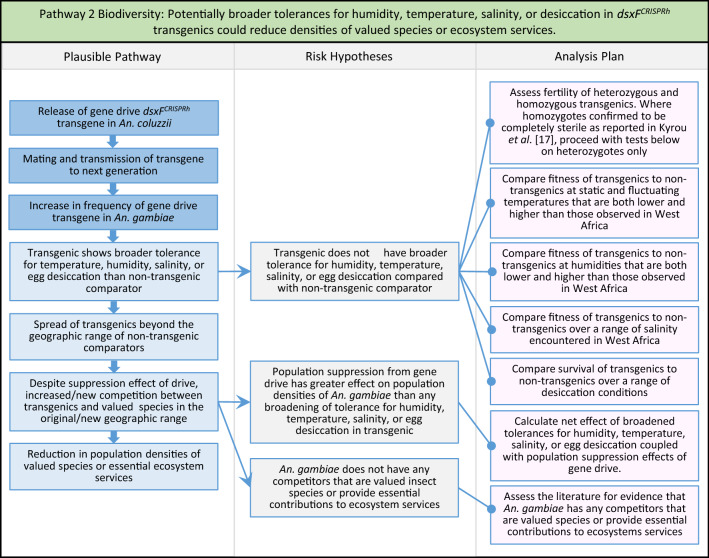
Pathway 2 Biodiversity: Potentially broader tolerances for humidity, temperature, salinity, or desiccation in *dsxF*^*CRISPRh*^ transgenics could reduce densities of valued species or ecosystem services. Were the transgenic to show a broadening of tolerance for environmental conditions, this could result in increased competition with existing species in its current range, as well as new competition with new species in new range. Transgenics with broadened tolerance for humidity and temperature could, for example, be expected to show extended survival into dry season compared to non-transgenic. The net effect of a population suppression gene drive could ultimately reduce this specific harm by reducing the density of mosquitoes, including transgenic ones

**Fig. 5:**
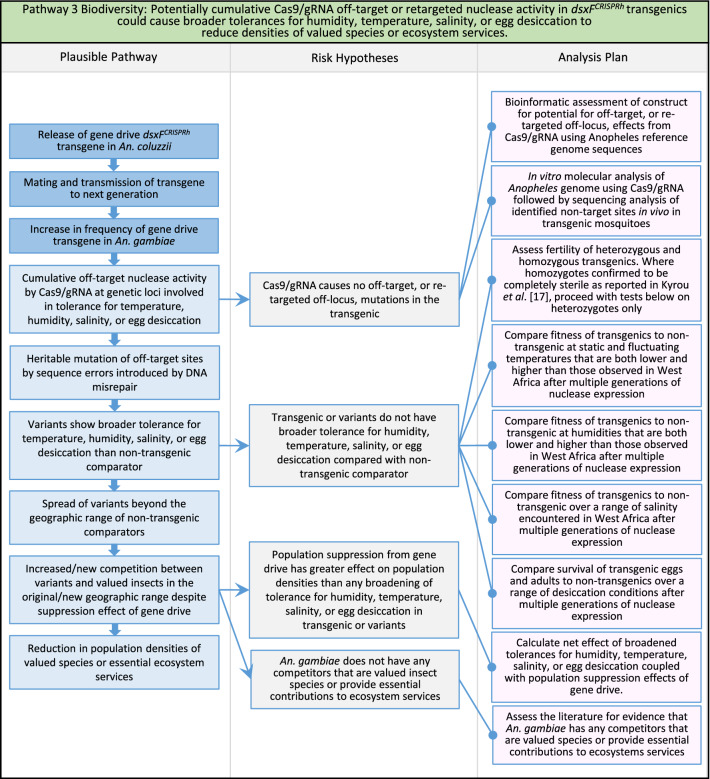
Pathway 3 Biodiversity: Potentially cumulative Cas9/gRNA off-target or retargeted nuclease activity in *dsxF*^*CRISPRh*^ transgenics could cause broader tolerances for humidity, temperature, salinity, or egg desiccation to reduce densities of valued species or ecosystem services. Were the transgenic to show off-target or retargeted mutations leading to a broadening of tolerance for environmental conditions, this could result in increased competition from variants with existing species in its current range, as well as new competition from variants with new species in new range. Variants with broadened tolerance for humidity and temperature could also show extended survival into dry season compared to non-transgenic. The net effect of a population suppression gene drive could ultimately reduce this specific harm by reducing the density of mosquitoes, including variants. For this pathway, the first tier of the analysis plan would involve bioinformatic and molecular assessments of the potential for off-target or retargeted mutations to occur in the transgenic. In the event of such mutations being detected, a second tier of phenotypic characterisations would then be performed

**Fig. 6:**
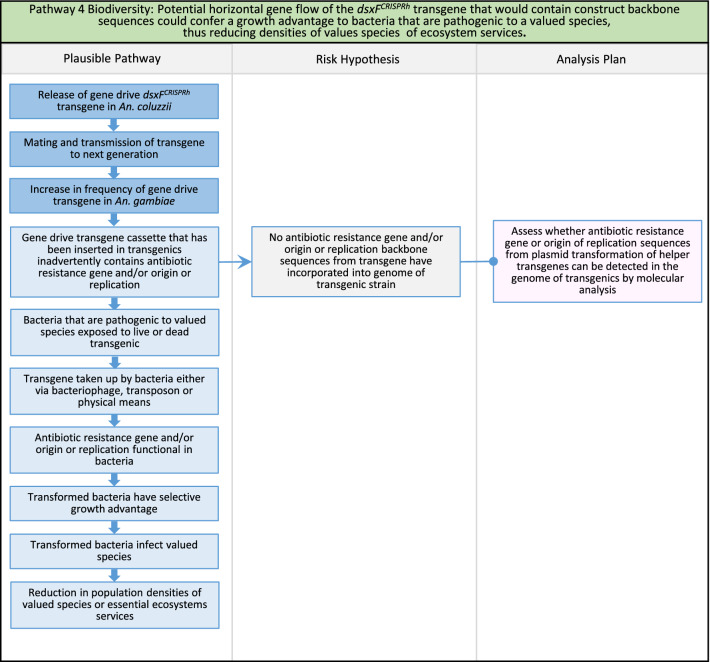
Pathway 4 Biodiversity: Potential horizontal gene flow of the *dsxF*^*CRISPRh*^ transgene that would contain construct backbone sequences could confer a growth advantage to bacteria that are pathogenic to a valued species, thus reducing densities of valued species or ecosystem services. Horizontal gene transfer is not a harm *per se*. However, the horizontal transfer of gene that provides some growth advantage to a prokaryote could represent a potential harm. In any event, the presence of antibiotic resistance genes from the backbone sequences of transformation construct containing the transgene should be detected during standard product development and cause the transgenic strain to be eliminated for further progress towards field release. Nonetheless, this pathway is included here for the sake of completeness

**Fig. 7:**
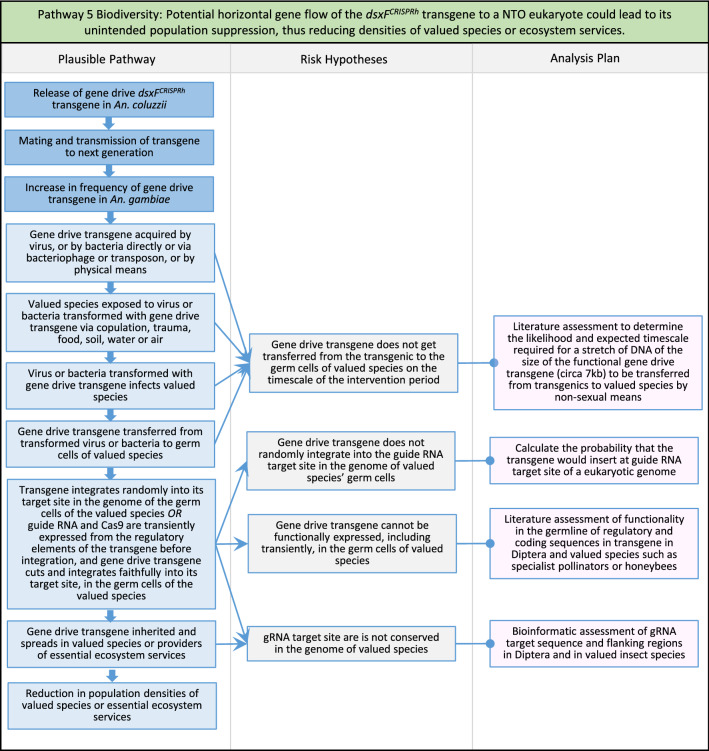
Pathway 5 Biodiversity: Potential horizontal gene flow of the *dsxF*^*CRISPRh*^ transgene to a NTO eukaryote could lead to its unintended population suppression, thus reducing densities of valued species or ecosystem services. The *An. gambiae* complex is considered to be made up of nine cryptic species, namely *An. amharicus, An. arabiensis, An. bwambae, An. coluzzii, An. fontenillei, An. gambiae s.s., An. melas, An. merus*, and *An. quadriannulatus* [48–52]. Because the guide RNA target sequence of the *dsxF*^*CRISPRh*^ transgene that are conserved in all of the above species examined [17], transfer of this transgene between any of these species via hybridization would likely lead to functional gene drive and population suppression in those species. Thus, all of the above species are considered TOs. The most closely related species to *An. gambiae* is *An. christyi* which differs morphologically, and is genetically distinct, from *An. gambiae*, with both species being separated by circa 9 million years of evolution [72]. The absence of observed gene flow between both species supports the lack of any significant hybridization between these species so that for even less closely-related species of *Anopheles* hybridization *with An. gambiae* is considered implausible. Hybridization is therefore not considered a plausible mechanism for transfer of the gene drive transgene from *An. gambiae* to NTOs including valued species

**Fig. 8:**
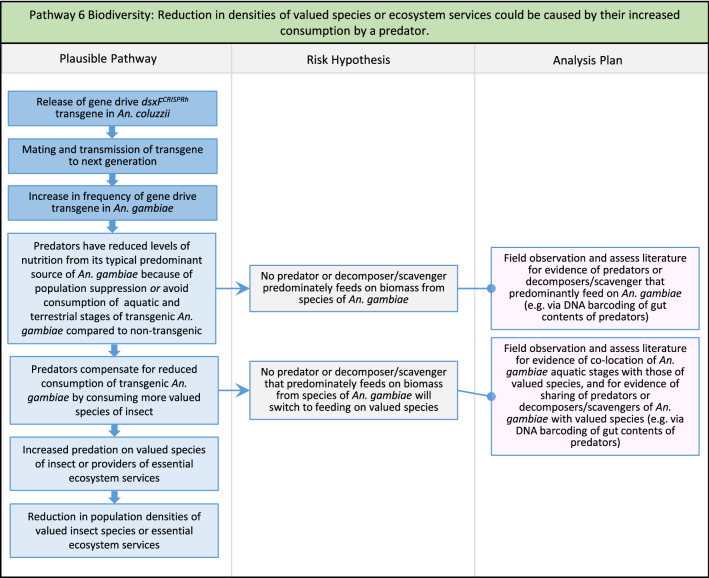
Pathway 6 Biodiversity: Reduction in densities of valued species or ecosystem services could be caused by their increased consumption by a predator. This is considered in the context of reductions in population density of valued species. It is likely to be most relevant in the setting of the aquatic habitat where *An. gambiae* larvae and pupae may constitute more significant food resources for predators, decomposers and scavengers than adult mosquitoes in terrestrial habitats [75]

**Fig. 9:**
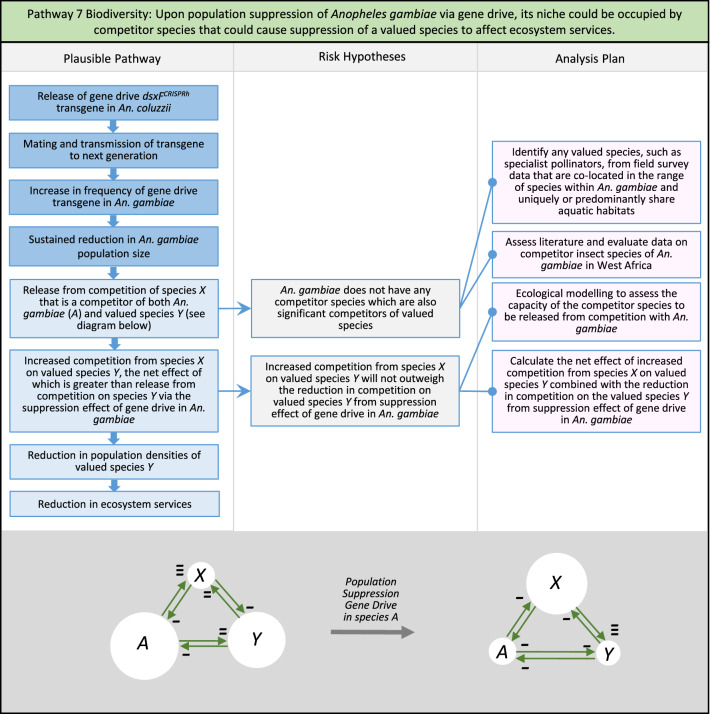
Pathway 7 Biodiversity: Upon population suppression of *Anopheles gambiae* via gene drive, its niche could be occupied by competitor species that could cause suppression of a valued species to affect ecosystem services. For this potential harm, the valued species could be reduced in density via two potentially countervailing effects (see illustration). *A*, *X* and *Y* denotes *An. gambiae*, species *X* and valued species *Y*, respectively. Size of white circles indicates notional population density of species. Green arrows indicate competitive pressure of one species on another, with - symbol representing negative effect on species at arrowhead, and - - or - - - indicating even greater negative effects. Firstly, species *X* could be released from competition with *A, An. gambiae*, following the impact of population suppression gene drive, which in turn could increase competition from species *X* on valued species *Y*. An additional possibility is that species *Y* could also be released from competition with *An. gambiae* which could act to increase the density of species *Y*. In that case, for the potential harm to occur the net effect of increased competition from species *X* with decreased competition from *An. gambiae* on species *Y* could still lead to the population of species *Y* being reduced

**Fig. 10:**
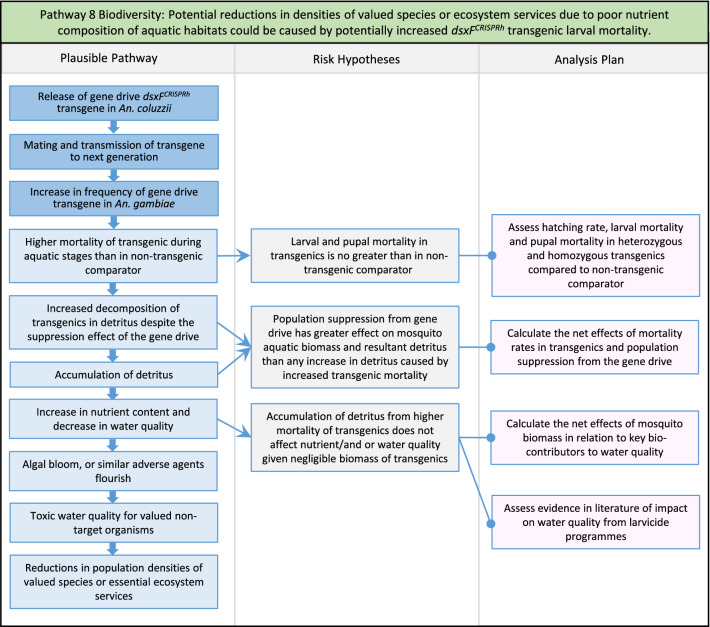
Pathway 8 Biodiversity: Potential reductions in densities of valued species or ecosystem services due to poor nutrient composition of aquatic habitats could be caused by potentially increased *dsxF*^*CRISPRh*^ transgenic larval mortality. A population suppression gene drive does not *a priori* have to result in higher mortality during aquatic stages. Therefore, this pathway is plausible without necessarily being likely. Indeed, the net effect from reductions in the density of aquatic stages caused by population suppression gene drive may be to reduce detritus. In addition, many species of the complex, especially *An. gambiae s.s.*, prefer aquatic habitats that contain clean water, are sunlit, lack vegetation and are ephemeral. They are thus unlikely to represent significant habitats for valued species

**Fig. 11:**
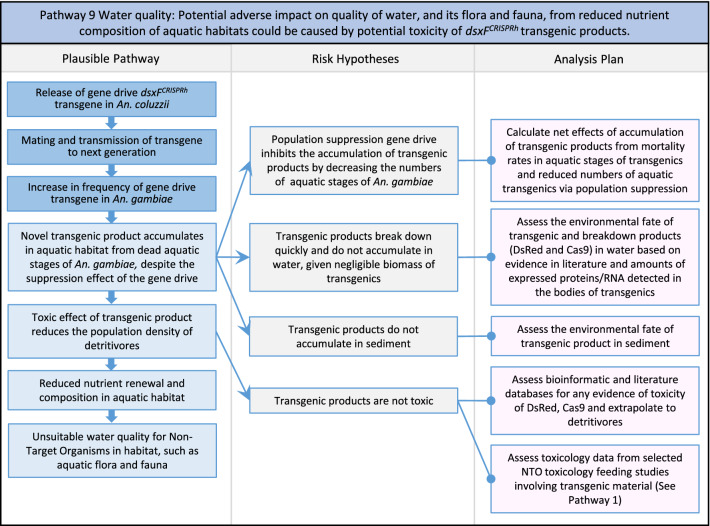
Pathway 9 Water quality: Potential adverse impact on quality of water, and its flora and fauna, from reduced nutrient composition of aquatic habitats could be caused by potential toxicity of *dsxF*^*CRISPRh*^ transgenic products. The net effect from reductions in the density of aquatic stages caused by a population suppression gene drive should be to reduce the overall density of aquatic stages of *An. gambiae*

**Fig. 12:**
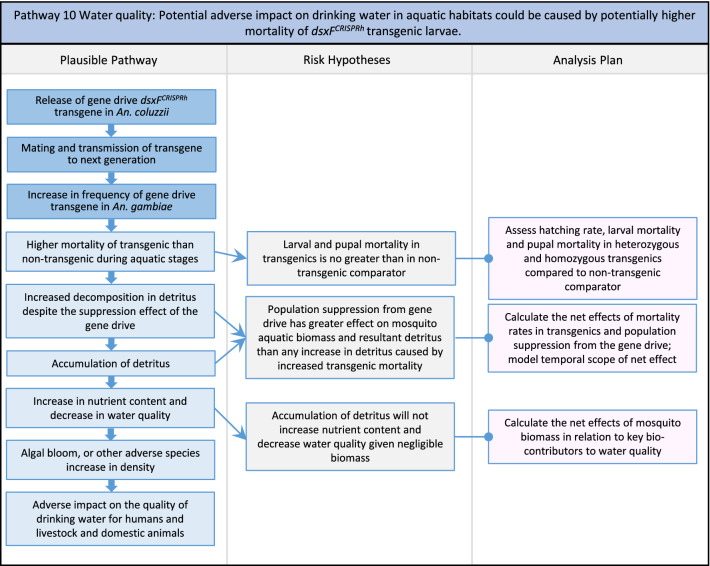
Pathway 10 Water quality: Potential adverse impact on drinking water in aquatic habitats could be caused by potentially higher mortality of *dsxF*^*CRISPRh*^ transgenic larvae. A population suppression gene drive does not *a priori* have to result in higher mortality during aquatic stages. Therefore, this pathway is plausible without necessarily being likely. Indeed, the net effect from reductions in the density of aquatic stages caused by population suppression gene drive may be to reduce detritus. In addition, many species of the complex, especially *An. gambiae s.s.*, prefer aquatic habitats that contain clean water, are sunlit, lack vegetation and are ephemeral. These habitats are thus unlikely to represent major sources of drinking water, particularly for humans

**Fig. 13:**
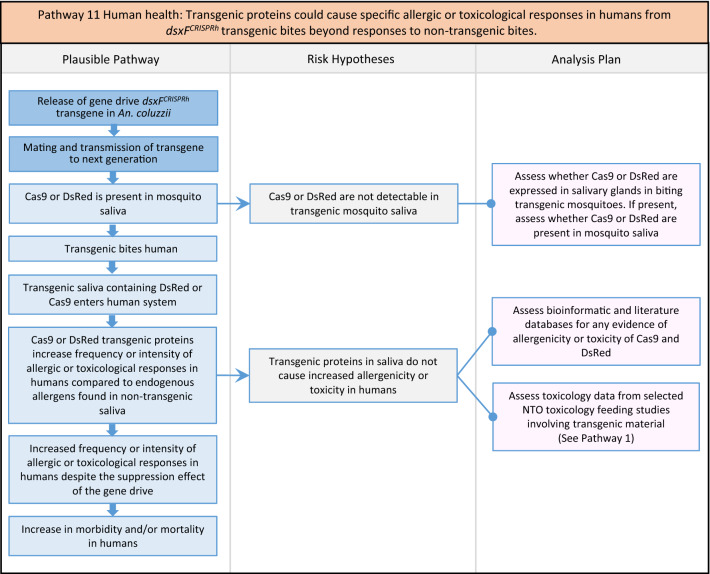
Pathway 11 Human health: Transgenic proteins could cause specific allergic or toxicological responses in humans from *dsxF* transgenic bites beyond responses to non-transgenic bites. For toxicological responses, manifestation of this potential harm would depend on (i) whether the transgenic proteins are toxic to humans; (ii) whether those proteins are expressed in the saliva of transgenics at doses known to be harmful to humans. Toxicity profiles of transgenic proteins could be informed by bioinformatics analyses and inferences from toxicology studies in NTOs. For allergic responses, this potential harm is based on the hazard to an individual human from exposure to the transgenic proteins in saliva from bites of transgenic mosquitoes

**Fig. 14:**
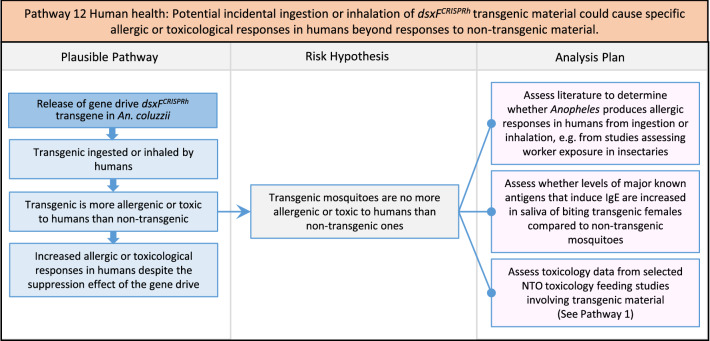
Pathway 12 Human health: Potential incidental ingestion or inhalation of *dsxF*^*CRISPRh*^ transgenic material could cause specific allergic or toxicological responses in humans beyond responses to non-transgenic material. The plausibility of this potential harm and its pathway is likely to rest on weight of evidence from literature [141] and analogous situations in programmes employing other species, e.g. SIT, rather than from definitive evidence from specific laboratory studies

**Fig. 15:**
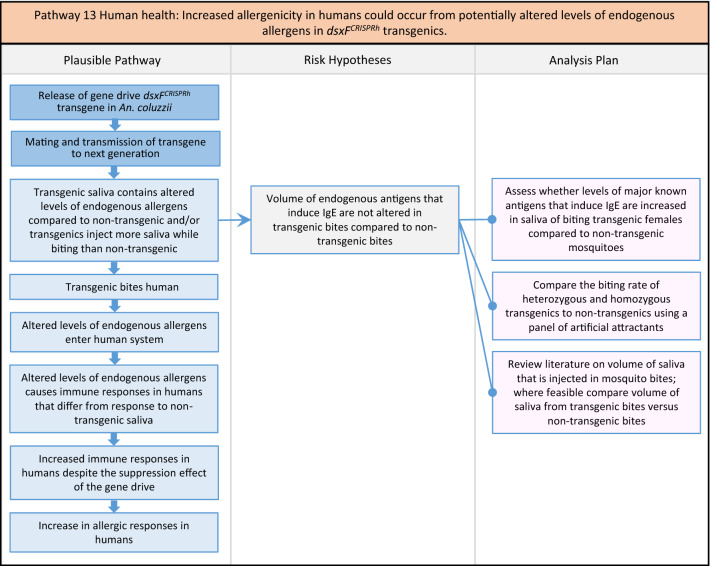
Pathway 13 Human health: Increased allergenicity in humans could occur from potentially altered levels of endogenous allergens in *dsxF*^*CRISPRh*^ transgenics. The plausible pathway here is based on the potential harm that would be caused to an individual human from exposure to altered levels of endogenous mosquito proteins in transgenics [141]

**Fig. 16:**
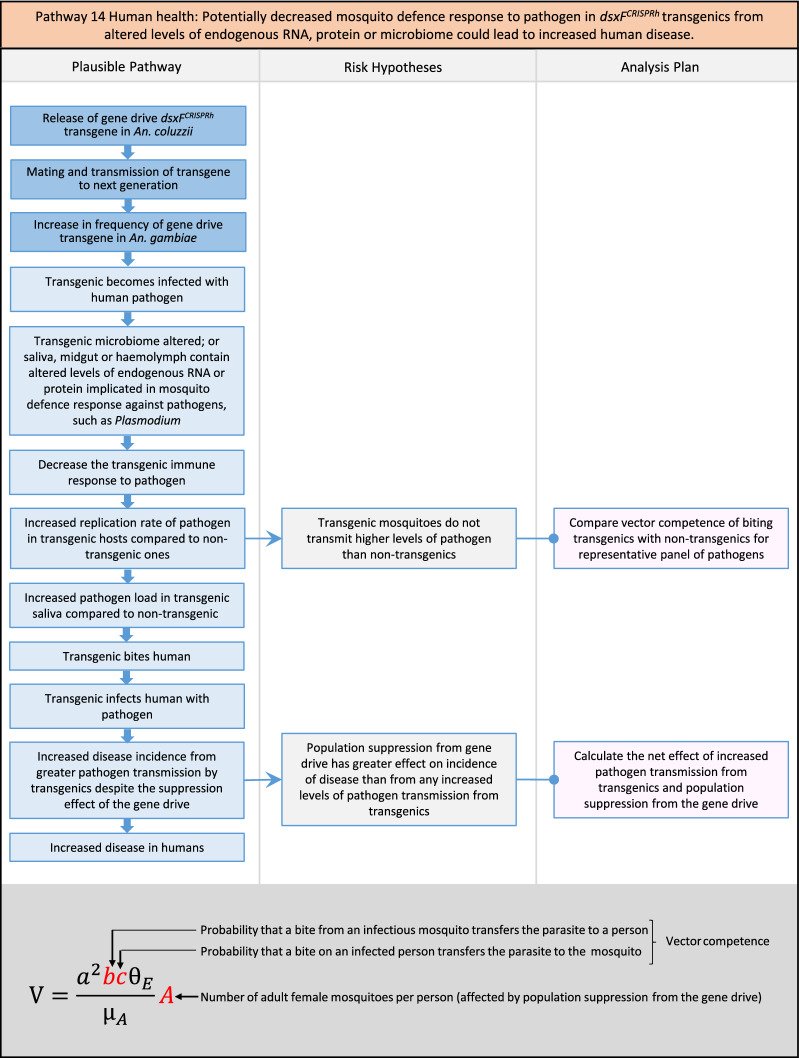
Pathway 14 Human health: Potentially decreased mosquito defence response to pathogen in *dsxF*^*CRISPRh*^ transgenics from altered levels of endogenous RNA, protein or microbiome could lead to increased human disease. Mosquito RNA or protein in saliva, midgut or haemolymph, or contents of the microbiome, can alter defence responses to pathogens such as *Plasmodium* or ONNV [112–116]. The components of vectorial capacity (V) that would be affected in this pathway are shown in red in the equation

**Fig. 17:**
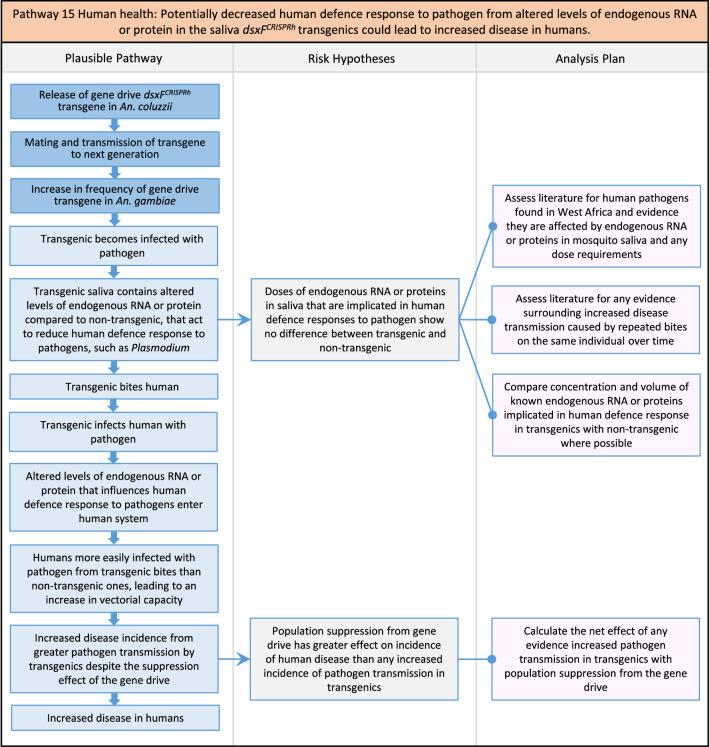
Pathway 15 Human health: Potentially decreased human defence response to pathogen from altered levels of endogenous RNA or protein in the saliva *dsxF*^*CRISPRh*^ transgenics could lead to increased disease in humans. The plausibility of this pathway stems from increasing evidence in the literature indicating that mosquito RNA injected from its saliva during biting might affect vector-host-parasite interactions [142–144]. The net effect of the population suppression gene drive could ultimately be to reduce this potential harm by reducing the density of mosquitoes including transgenic ones

**Fig. 18:**
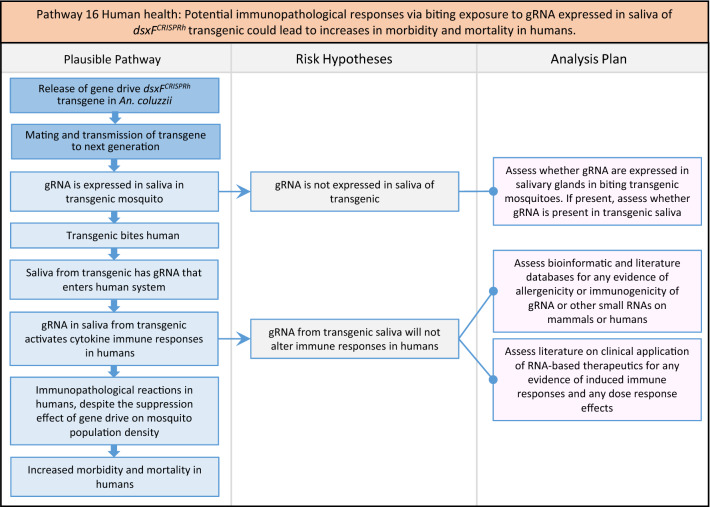
Pathway 16 Human health: Potential immunopathological responses via biting exposure to gRNA expressed in saliva of *dsxF*^*CRISPRh*^ transgenic could lead to increases in morbidity and mortality in humans. As the gRNA in the gene drive cassette is expressed ubiquitously and constitutively from the U6 promoter, it could be present in the saliva of transgenics. *In vitro* transcribed gRNA has been reported to induce strong expression of cytokines and cytotoxicity [145–147]. Induction of such cytokines from exposure to gRNA in humans could lead to immunopathological reactions such as aberrant inflammatory responses resulting in excessive pain, pyrogenic fever, inflammation and tissue damage, potentially increasing morbidity and mortality [148]. The net effect of the population suppression gene drive could ultimately be to reduce this potential harm by reducing the density of mosquitoes including transgenic ones

**Fig. 19:**
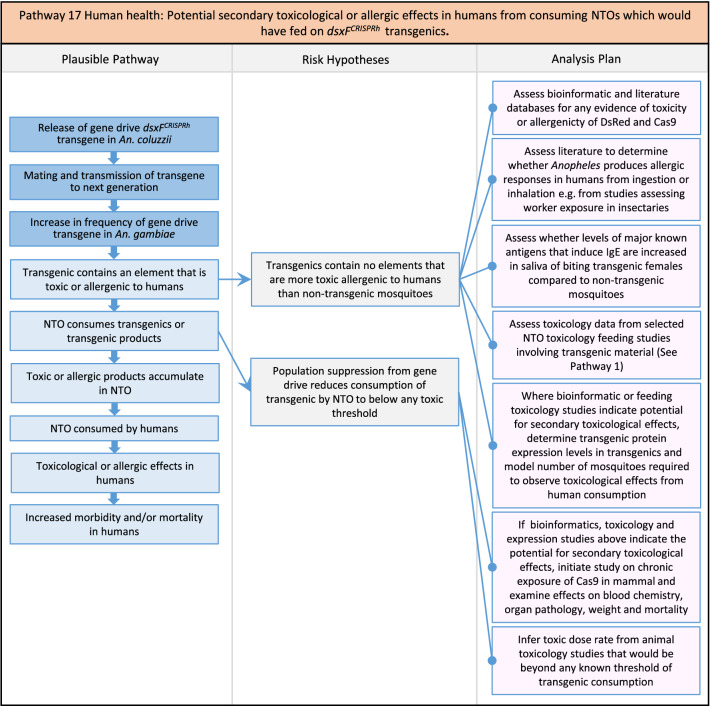
Pathway 17 Human health: Potential secondary toxicological effects in humans from consuming NTOs which would have fed on *dsxF*^*CRISPRh*^ transgenics. The analysis plan draws on the weight of evidence from toxicity studies on DsRed and Cas9, as well as toxicology studies on indicator species outlined in Pathway 1 (Fig. 3). Given body mass ratio of humans compared to transgenic mosquitoes, it was considered implausible that the transgenic would be toxic to humans but not the NTOs on which humans might feed, having a reduced body mass ratio compared to transgenic mosquitoes. Where the weight of evidence on secondary toxicological effects remains equivocal, then further experimental studies in an indicator species of mammal, such as the rat, could be pursued in a tiered analysis plan. The net effect of the population suppression gene drive could ultimately be to reduce this potential harm by reducing the density of mosquitoes including transgenic ones and therefore any potential for NTO feeding on transgenics

**Fig. 20:**
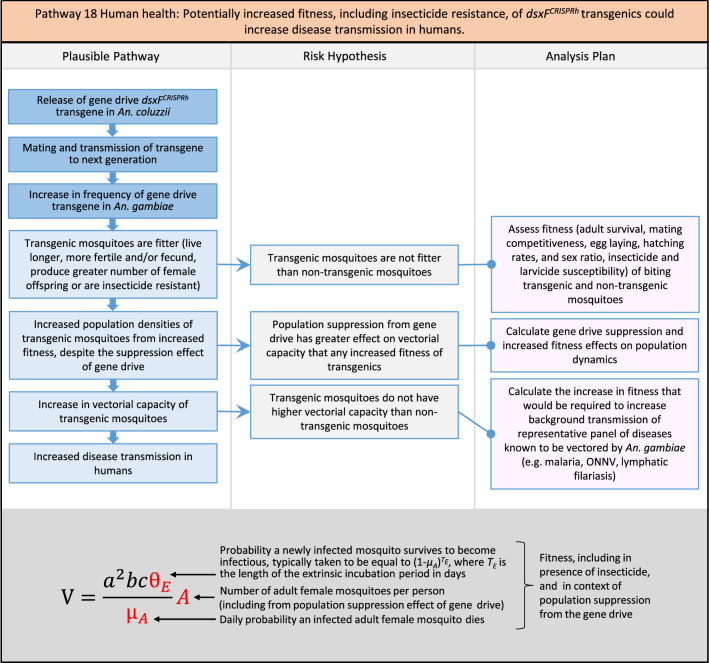
Pathway 18 Human health: Potentially increased fitness, including insecticide resistance, of *dsxF*^*CRISPRh*^ transgenics could increase disease transmission in humans. For this analysis plan, the measurement endpoints for relevant fitness parameters would be female lifespan, population density and sex ratio, and fecundity and insecticide resistance in females. Although technically a plausible pathway to potential harm identified by problem formulation, increased fitness of the transgenic should be detected during standard product development as it would most likely be considered a major product failure for most transgenic strains and cause the strain to be eliminated for further progress towards field release. Nonetheless, this potential harm is included here for the sake of completeness. The net effect of the population suppression gene drive could ultimately be to reduce this potential harm by reducing the density of mosquitoes including transgenic ones. The components of vectorial capacity (V) that would be affected in this pathway are shown in red in the equation

**Fig. 21:**
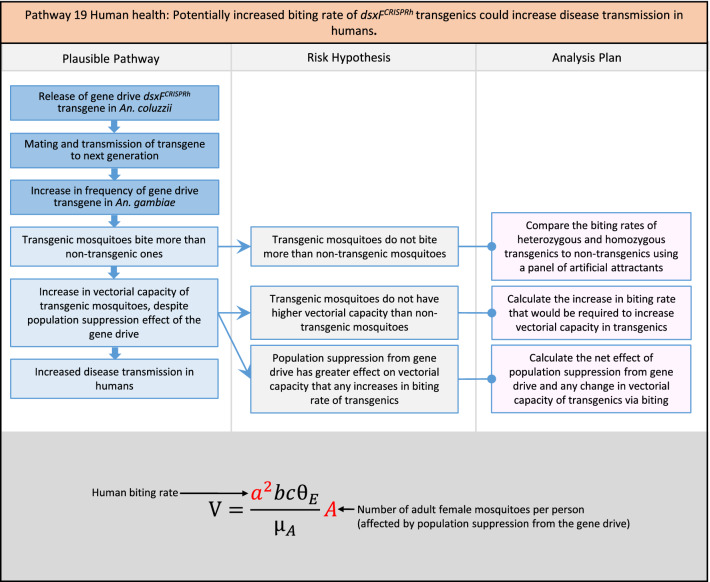
Pathway 19 Human health: Potentially increased biting rate of *dsxF*^*CRISPRh*^ transgenics could increase disease transmission in humans. Although increased biting on humans (*a*^*2*^) could lead to increases in vectorial capacity, overall the net effect of the population suppression gene drive could ultimately be to reduce this potential harm by reducing the density of mosquitoes (*A*) including transgenic ones. The components of vectorial capacity (V) that would be affected in this pathway are shown in red in the equation

**Fig. 22:**
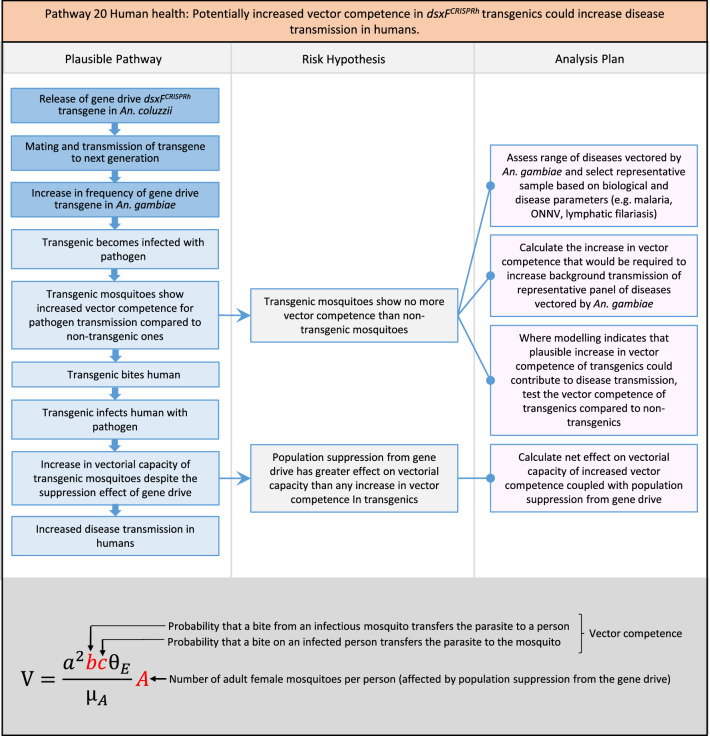
Pathway 20 Human health: Potentially increased vector competence in *dsxF*^*CRISPRh*^ transgenics could increase disease transmission in humans. While increased vector competence (*bc*) would lead to increases in vectorial capacity, the net effect of a population suppression gene drive could ultimately reduce the impact of this potential harm by reducing the density of mosquitoes (*A*), including transgenic ones. Alternatively, vector competence could increase as a result of decreased densities of *An. gambiae* [149, 150], which would be assessed via the modelling outlined in steps two and four of the analysis plan. The components of vectorial capacity (V) that would be affected in this pathway are shown in red in the equation

**Fig. 23:**
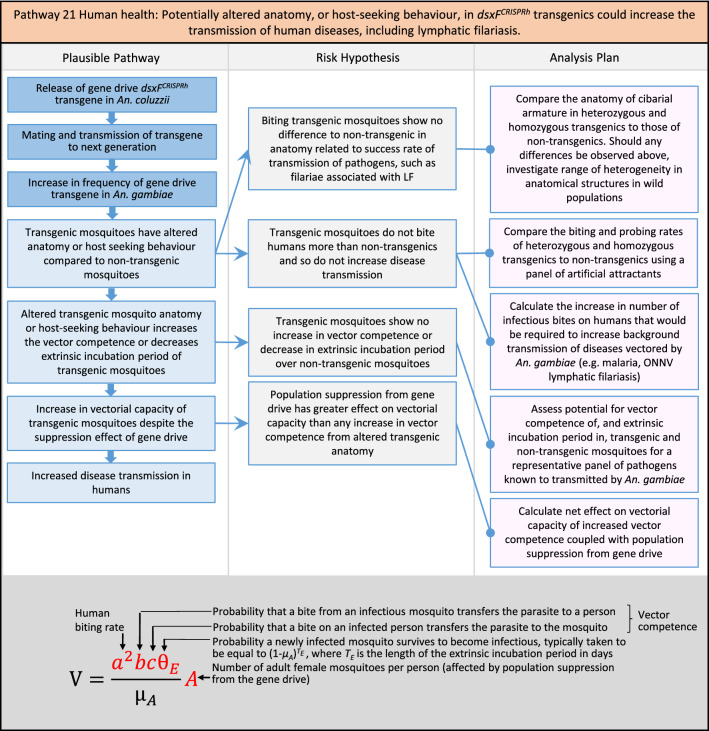
Pathway 21 Human health: Potentially altered anatomy, or host-seeking behaviour, in *dsxF*^*CRISPRh*^ transgenics could increase the transmission of human diseases, including lymphatic filariasis (LF). This pathway is about the efficiency of transmission, so any change in anatomical characteristics in the transgenics may increase the biting or probing rate or might increase the transmission rate from a given biting rate. Host seeking behavioural alteration is defined here as preferences for sources of blood meals based primarily on olfactory cues. After malaria, lymphatic filariasis is the most burdensome disease transmitted by species of *An. gambiae.* For example, in Burkina Faso, Malaria accounts for 17.96 % of total DALYs vs. 0.29 % for total DALYs for LF, 0.05 % of total DALYs for yellow fever and 0.0094 % of total DALYs for dengue [101]. Direct laboratory assessment of the vector competence of *An. gambiae* for the most common LF parasite *Wuchereria bancrofti* has not reliably been established [102]. However, as *dsxF*^*CRISPRh*^ transgenic mosquitoes have reported anatomical alterations [17], and the cibarial armature is implicated in transmission of LF to mosquitoes, its anatomy should be examined in transgenics [103–105]. The components of vectorial capacity (V) that would be affected in this pathway are shown in red in the equation

**Fig. 24:**
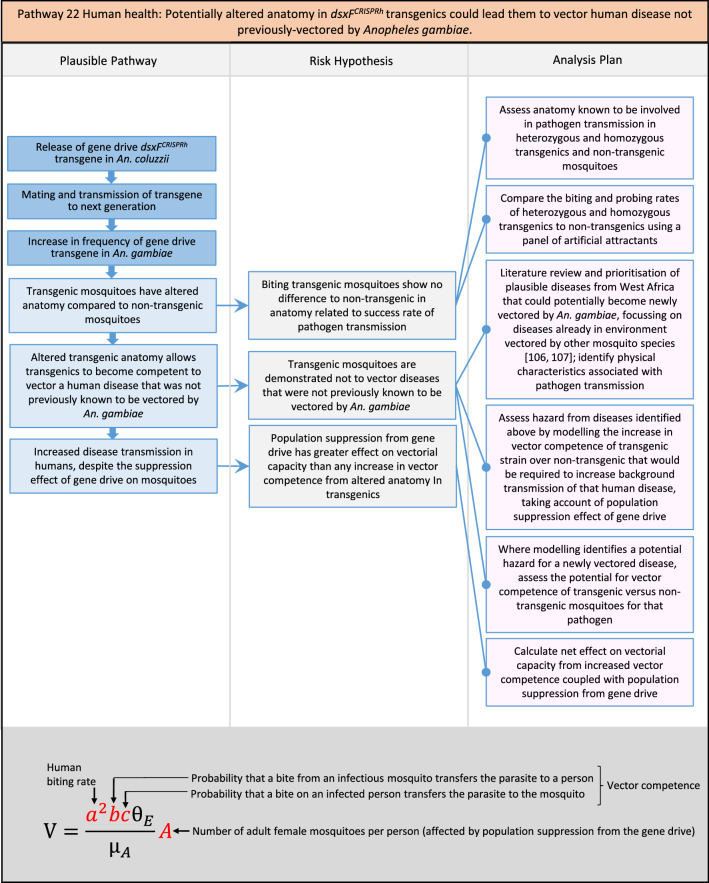
Pathway 22 Human health: Potentially altered anatomy in *dsxF*^*CRISPRh*^ transgenics could lead them to vector human disease not previously vectored by* Anopheles gambiae*. This pathway is about the efficiency of transmission, so any change in anatomical characteristics in the transgenics may increase the biting or probing rates or might increase the disease transmission rates. As *dsxF*^*CRISPRh*^ transgenic mosquitoes have reported anatomical alterations [17], the analysis plan includes examination of anatomical structures implicated in disease transmission [102]. The potential harm outlined here envisages a ‘worst case scenario’ where there would be increased disease in humans caused by increased vectorial capacity, despite decreases in overall mosquito densities via population suppression from the gene drive. The components of vectorial capacity (V) that would be affected in this pathway are shown in red in the equation

**Fig. 25:**
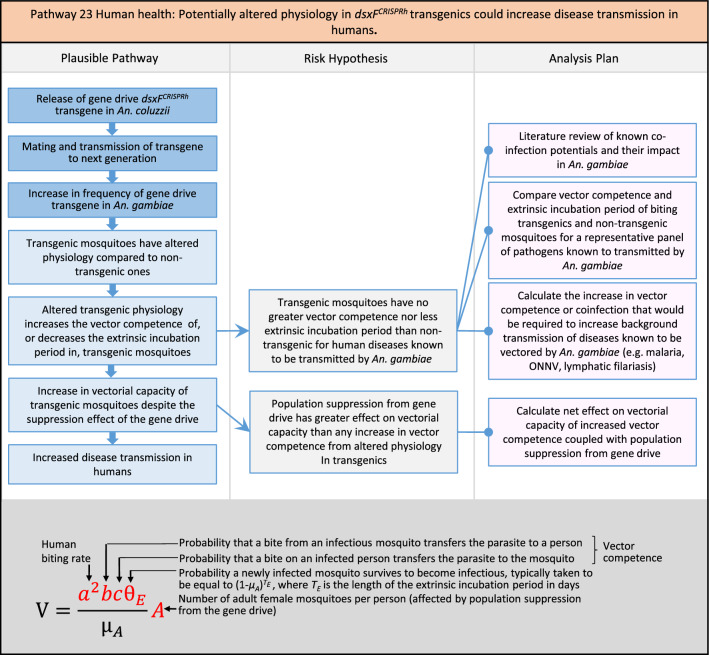
Pathway 23 Human health: Potentially altered physiology in *dsxF*^*CRISPRh*^ transgenics could increase disease transmission in humans. This pathway is about the efficiency of disease transmission, so any altered physiological characteristics in the transgenics compared to the non-transgenic, such as immune system function or capacity for co-infection or extrinsic incubation period [151], might increase the transmission rate of a given pathogen. The net effect of a population suppression gene drive would ultimately reduce the impact of this potential harm by reducing the density of mosquitoes, including transgenic ones. The components of vectorial capacity (V) that would be affected in this pathway are shown in red in the equation

**Fig. 26:**
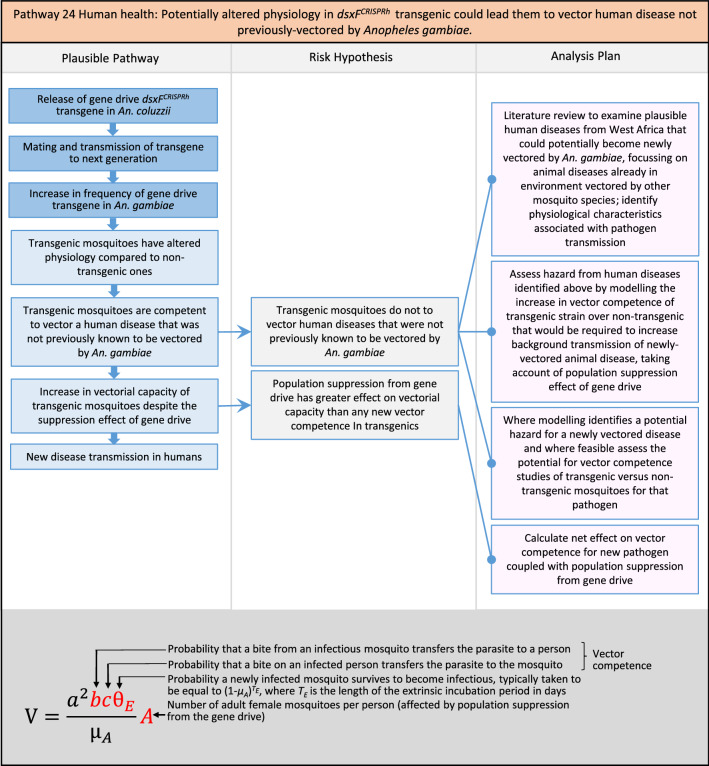
Pathway 24 Human health: Potentially altered physiology in *dsxF*^*CRISPRh*^ transgenic could lead them to vector human disease not previously vectored by* Anopheles gambiae*. Relevant physiology in this pathway might include immune system function or capacity for co-infection. Diseases newly transmitted by transgenics would necessarily already be present in the environment into which the transgenics were released. The components of vectorial capacity (V) that would be affected in this pathway are shown in red in the equation

**Fig. 27:**
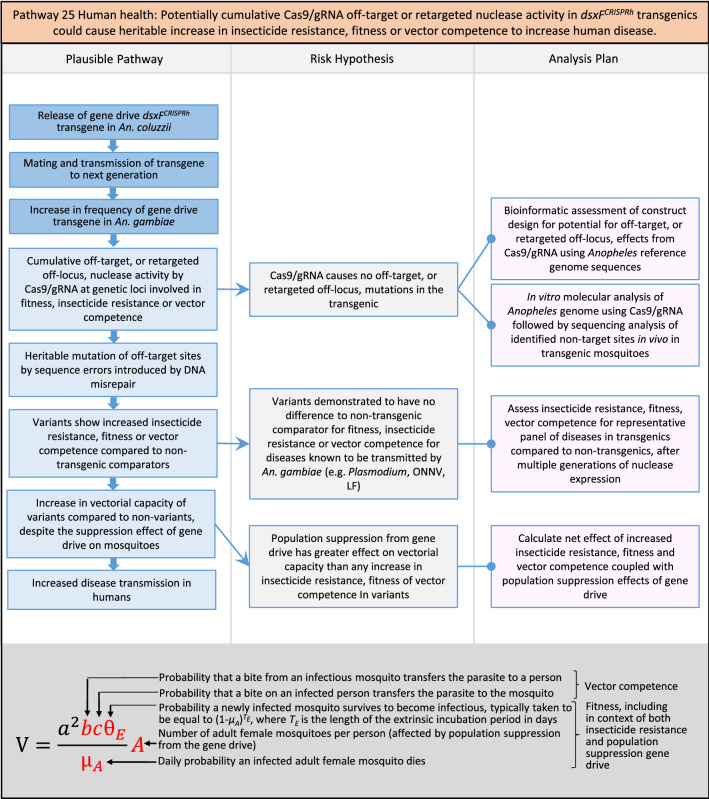
Pathway 25 Human health: Potentially cumulative Cas9/gRNA off-target or retargeted nuclease activity in *dsxF*^*CRISPRh*^ transgenics could cause heritable increase in insecticide resistance, fitness or vector competence to increase human disease. The net effect of the population suppression gene drive should ultimately be to reduce this specific harm by reducing the density of mosquitoes including transgenic ones. For this pathway, the first tier of the analysis would involve bioinformatic and molecular assessments of the potential for off-target or retargeted mutations to occur in the transgenic. In the event of such mutations being detected, a second tier of phenotypic characterizations would then be performed. The components of vectorial capacity (V) that would be affected in this pathway are shown in red in the equation

**Fig. 28:**
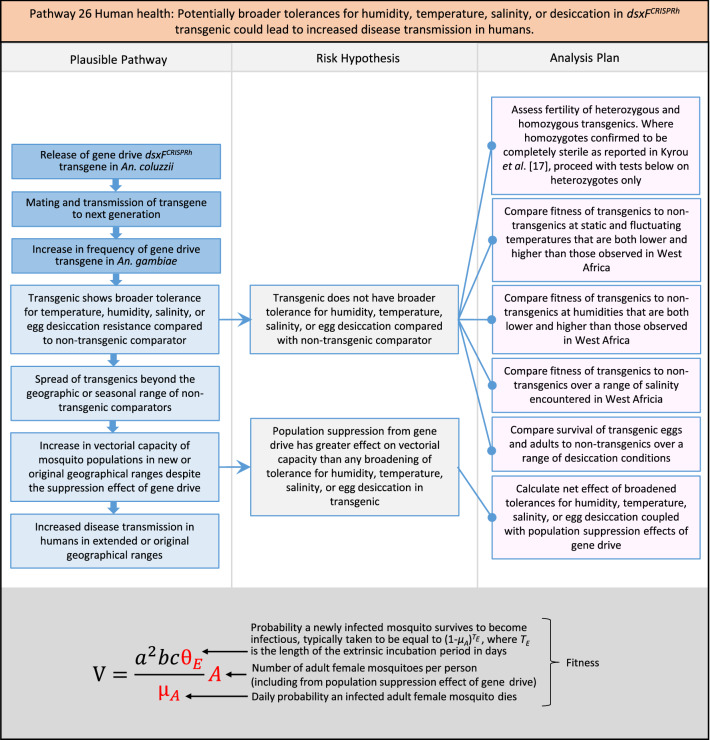
Pathway 26 Human health: Potentially broader tolerances for humidity, temperature, salinity, or desiccation in *dsxF*^*CRISPRh*^ transgenic could lead to increased disease transmission in humans. Were the transgenic to show a broadening of tolerance for environmental conditions, this could result in increased competition with existing species in its current geographic range, as well as new competition with new species in new range, in each case potentially increasing disease transmission. Transgenics with broadened tolerance for humidity and temperature might, for example, be expected to show extended survival into dry season compared to non-transgenic. The net effect of a population suppression gene drive would ultimately reduce the impact of this potential harm by reducing the density of mosquitoes (*A*), including transgenic ones. The components of vectorial capacity (V) that would be affected in this pathway are shown in red in the equation

**Fig. 29:**
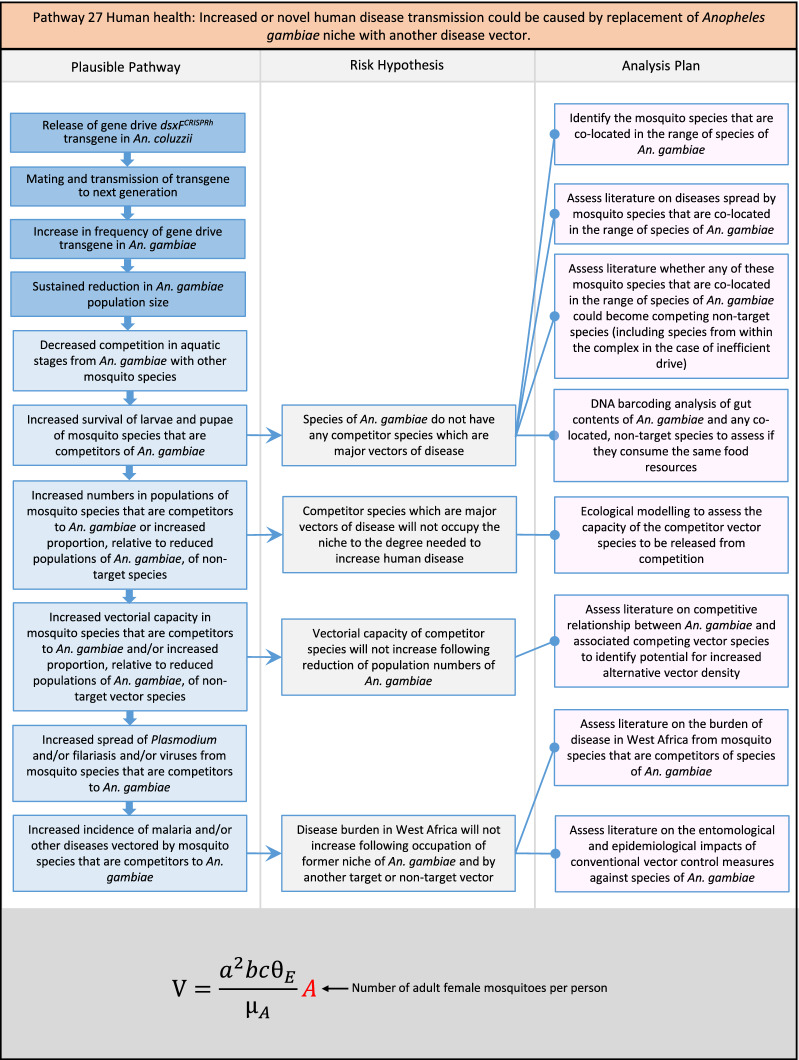
Pathway 27 Human health: Increased or novel human disease transmission could be caused by replacement of *Anopheles gambiae* niche with another disease vector. Population suppression of *An. gambiae* could release other disease vectors from competition, leading to an increase the population density of those other disease vectors and therefore increase their vectorial capacity and disease transmission. The components of vectorial capacity (V) that would be affected in this pathway are shown in red in the equation

**Fig. 30:**
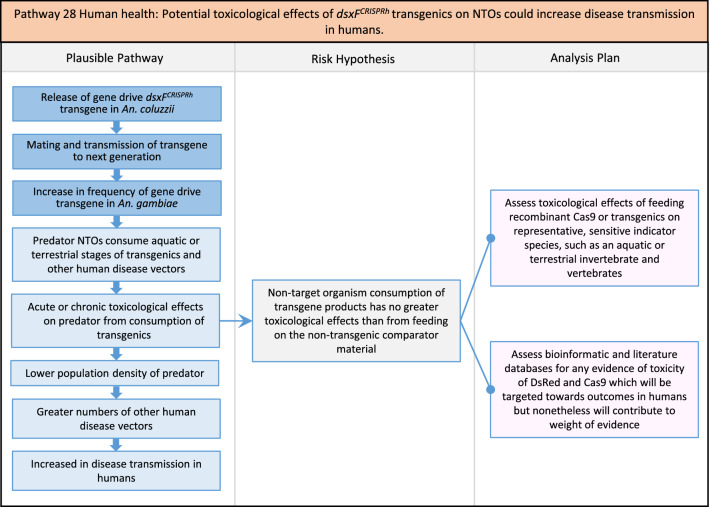
Pathway 28 Human health: Potential toxicological effects of *dsxF*^*CRISPRh*^ transgenics on NTOs could increase disease transmission in humans. Bioinformatic and literature evidence of any toxicity of DsRed and Cas9 will likely be more targeted towards outcomes in humans but nonetheless will contribute to weight of evidence supporting or refuting this pathway, whilst recognising that the mass of humans would greatly exceed that of any NTO. Defining the experimental conditions and choices of indicator species for chronic and acute studies will most likely involve discussion with national regulators, with reference to international regulatory guidance and best practice [26, 95]

**Fig. 31:**
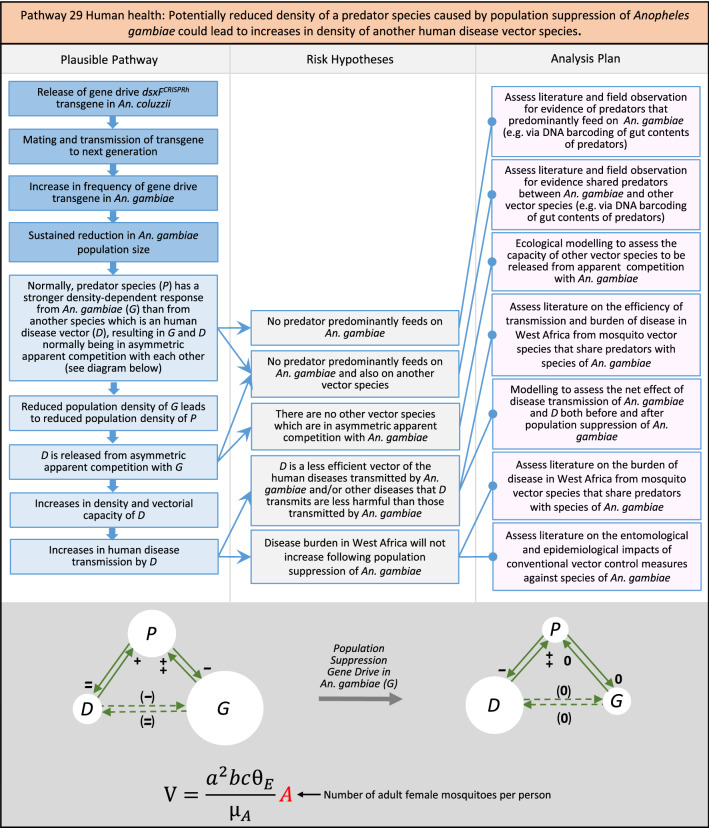
Pathway 29 Human health: Potentially reduced density of a predator species caused by population suppression of *Anopheles gambiae* could lead to increases in density of another human disease vector species. Species *P* denotes a predator of both *An. gambiae* (*G*) and another human disease vector species, *D*. Solid green lines represent direct effects of one species on another. Dashed green lines indicate indirect effects from apparent competition [109]. Size of white circles denotes notional size of species populations. The + symbol denotes a positive effect on the species at the arrowhead, with + + indicating stronger positive effects. The - symbol denotes a negative effect on the species at the arrowhead, with - - indicating stronger negative effects. 0 denotes a negligible effect on the species at the arrowhead. When symbols are within parentheses, this denotes an indirect effect on the species at the arrowhead. In this pathway, the asymmetric apparent competition between *G* and *D* (for example, see Fig. 1b in [109]), before population suppression gene drive is introduced in *G*, is lost following population suppression gene drive introduction, leading to reductions in the density of *P* and increases in the density of *D*. Sustained reduction in the population of *An. gambiae* could also lead to reduction in the density of a valued predator if that predator would, for example, feed predominantly on *An. gambiae* in the wet season and then switch to feeding on, and controlling the numbers of, another disease vector or pest in the dry season, when *An. gambiae* would not typically act as its predominant food source during that period. The components of vectorial capacity (V) that would be affected in this pathway are shown in red in the equation

**Fig. 32:**
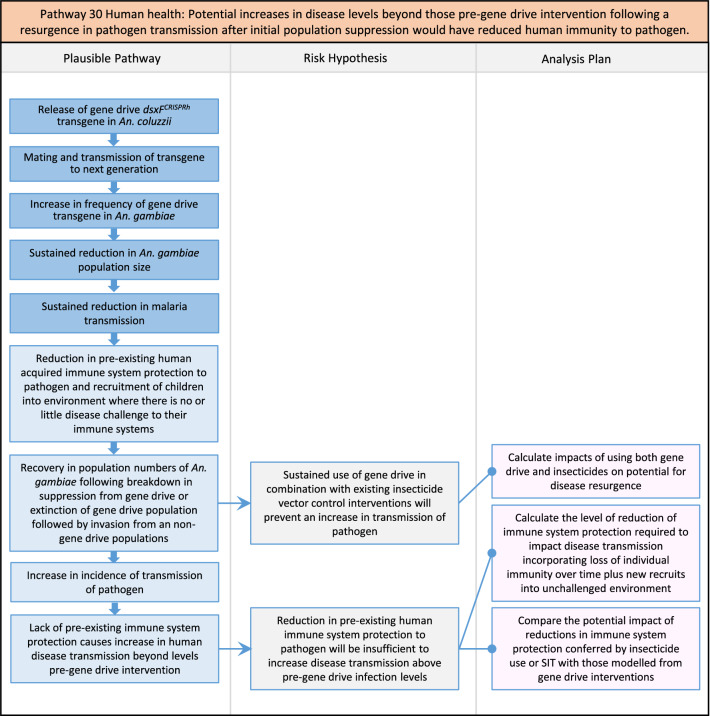
Pathway 30 Human health: Potential increases in disease levels beyond those pre-gene drive intervention following a resurgence in pathogen transmission after initial population suppression would have reduced human immunity to pathogen. This could be a potential harm for any potentially successful vector control agent, not just population suppression gene drive, but is included for the sake of completeness [152]. The analyses here are likely to lead to a ‘weight of evidence’ either in favour of or against the potential harm occurring

**Fig. 33:**
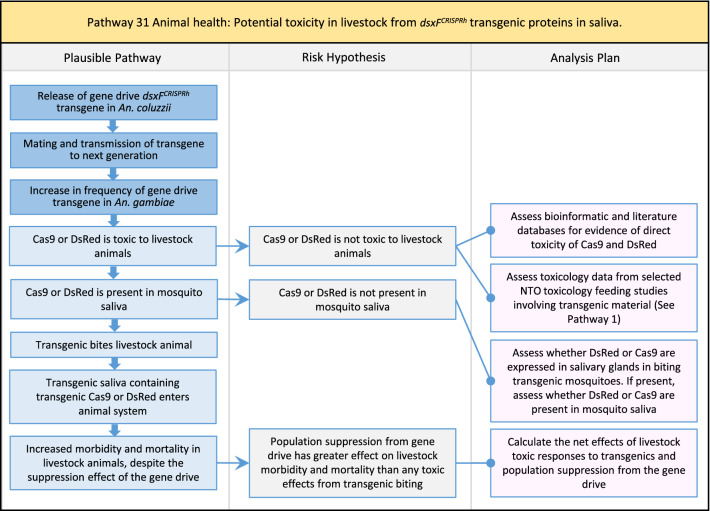
Pathway 31 Animal health: Potential toxicity in livestock from *dsxF*^*CRISPRh*^ transgenic proteins in saliva. This pathway is based on the potential harm that would be caused to an individual animal from exposure to the transgenic proteins. Manifestation of this potential harm would depend on (i) whether the transgenic proteins were toxic to livestock animals, and (ii) whether those proteins were expressed in the saliva of transgenics at doses known to be harmful to livestock animals. Toxicity profiles of transgenic proteins can be informed by bioinformatics analyses and inferences from toxicology studies in NTOs. The net effect of a population suppression gene drive would ultimately reduce this specific harm by reducing the density of the target species of mosquitoes, including transgenic ones

**Fig. 34:**
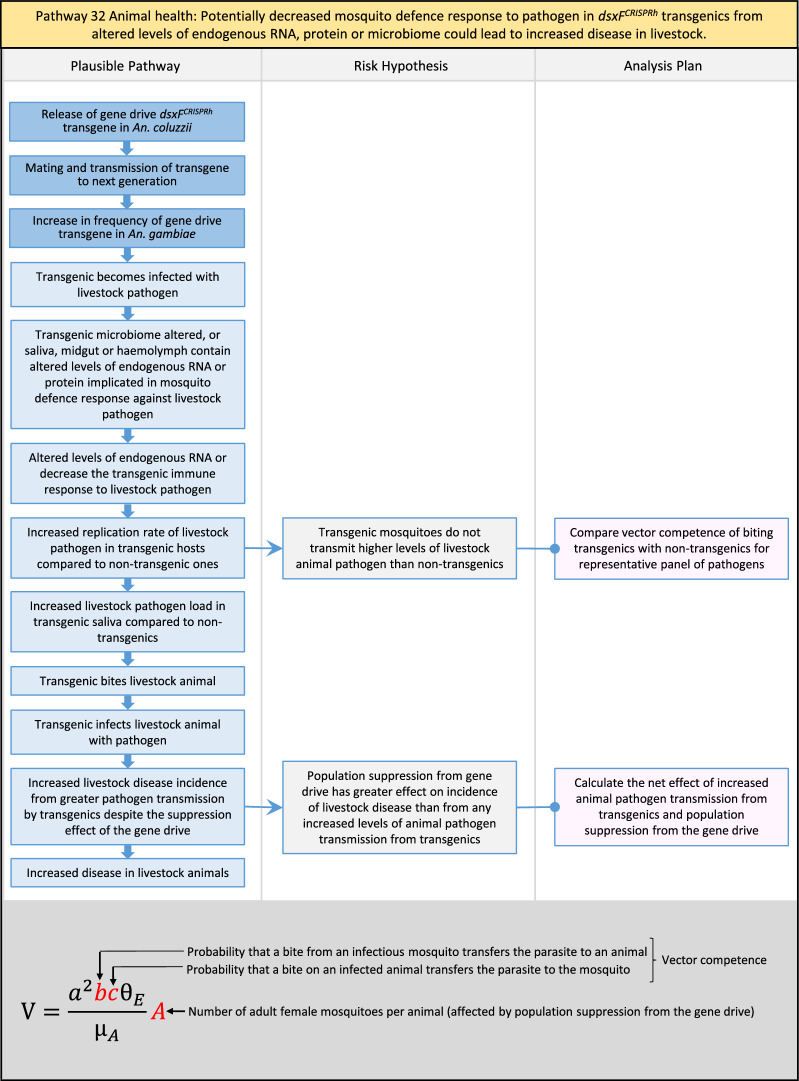
Pathway 32 Animal health: Potentially decreased mosquito defence response to pathogen in *dsxF*^*CRISPRh*^ transgenics from altered levels of endogenous RNA, protein or microbiome could lead to increased disease in livestock. Mosquito RNA or protein in saliva, midgut or haemolymph, or contents of the microbiome, can alter defence responses to human pathogens such as *Plasmodium* or ONNV [112–116]. The components of vectorial capacity (V) that would be affected in this pathway are shown in red in the equation

**Fig. 35:**
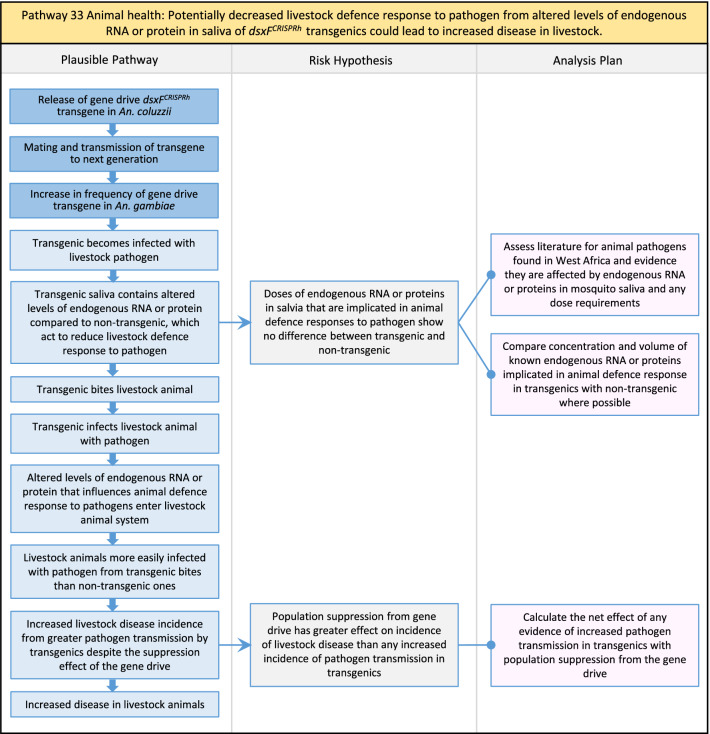
Pathway 33 Animal health: Potentially decreased livestock defence response to pathogen from altered levels of endogenous RNA or protein in saliva of *dsxF*^*CRISPRh*^ transgenics could lead to increased disease in livestock. Mosquito RNA injected from its saliva during biting could affect vector-host-parasite interactions [142–144]. The net effect of a population suppression gene drive would ultimately reduce this specific harm by reducing the density of the target species of mosquitoes, including transgenic ones

**Fig. 36:**
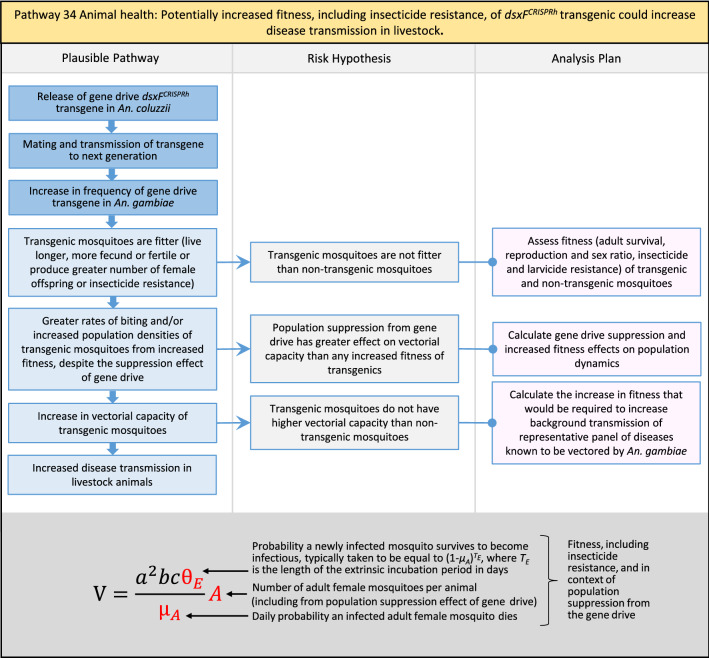
Pathway 34 Animal health: Potentially increased fitness, including insecticide resistance, of *dsxF*^*CRISPRh*^ transgenic could increase disease transmission in livestock. The net effect of the population suppression gene drive should ultimately be to reduce this specific harm by reducing the density of mosquitoes including transgenic ones. The components of vectorial capacity (V) that would be affected in this pathway are shown in red in the equation

**Fig. 37:**
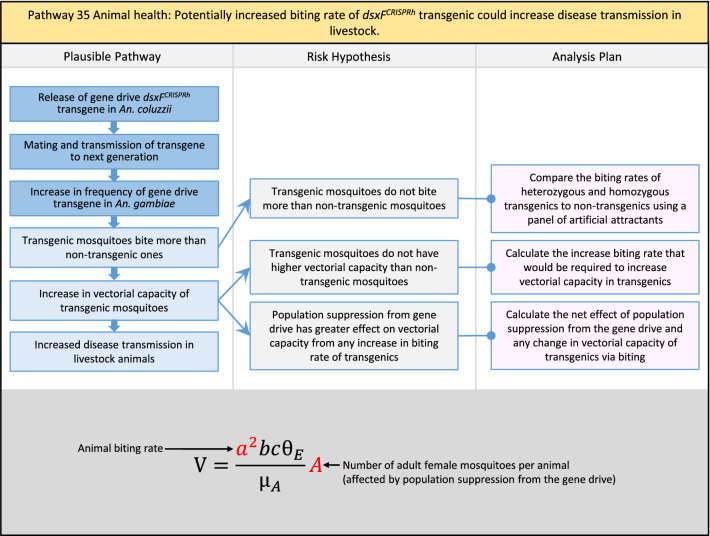
Pathway 35 Animal health: Potentially increased biting rate of *dsxF*^*CRISPRh*^ transgenic could increase disease transmission in livestock. The net effect of the population suppression gene drive should ultimately be to reduce this specific harm by reducing the density of mosquitoes including transgenic ones. The components of vectorial capacity (V) that would be affected in this pathway are shown in red in the equation

**Fig. 38:**
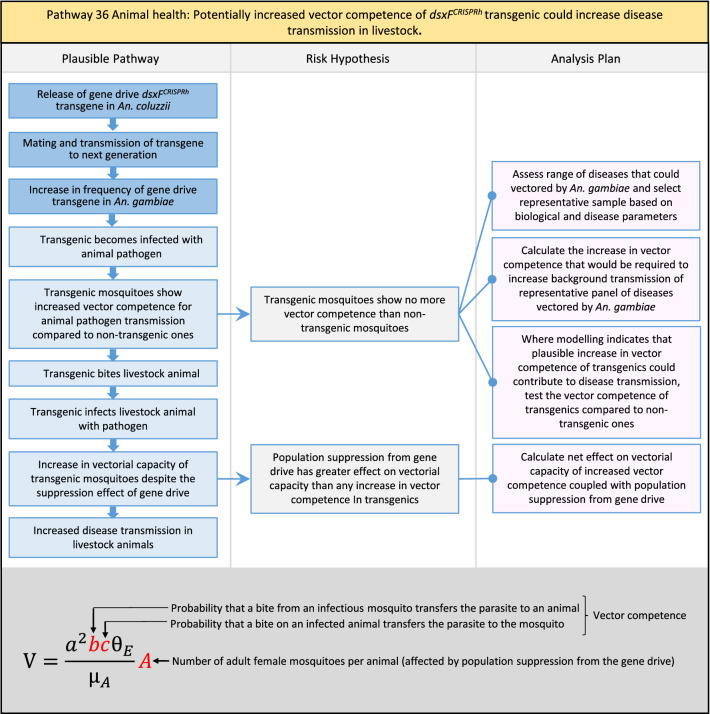
Pathway 36 Animal health: Potentially increased vector competence of *dsxF*^*CRISPRh*^ transgenic could increase disease transmission in livestock. The net effect of the population suppression gene drive should ultimately be to reduce this specific harm by reducing the density of mosquitoes including transgenic ones. The components of vectorial capacity (V) that would be affected in this pathway are shown in red in the equation

**Fig. 39:**
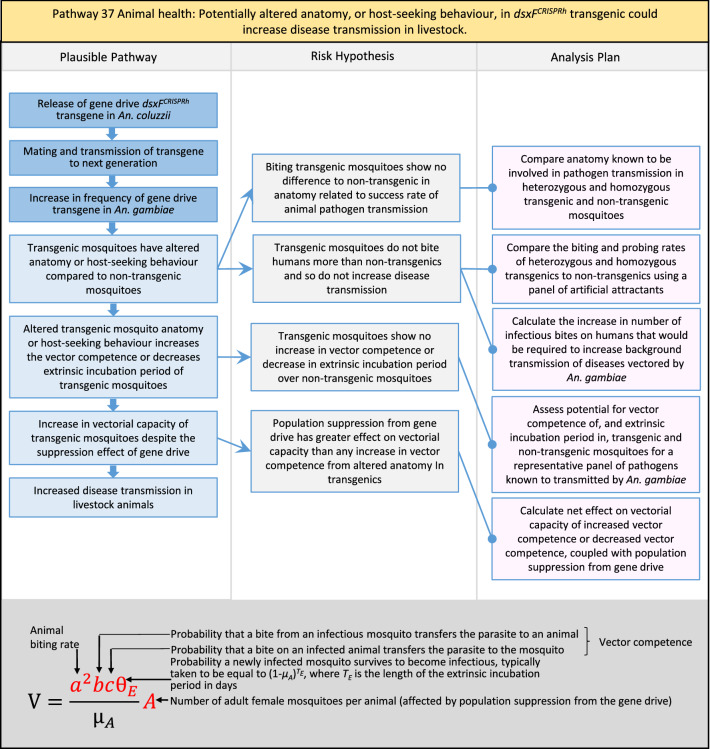
Pathway 37 Animal health: Potentially altered anatomy, or host-seeking behaviour, in *dsxF*^*CRISPRh*^ transgenic could increase disease transmission in livestock. This pathway is about the efficiency of disease transmission, so any change in anatomical characteristics in the transgenics could increase the biting or probing rates or disease transmission rates. As *dsxF*^*CRISPRh*^ transgenic mosquitoes have reported anatomical alterations [17], anatomical structures implicated in disease transmission, such as the cibarial armature, can be examined in transgenics [102]. The net effect of a population suppression gene drive would ultimately reduce the impact of this specific harm by reducing the density of mosquitoes, including transgenic ones. The components of vectorial capacity (V) that would be affected in this pathway are shown in red in the equation

**Fig. 40:**
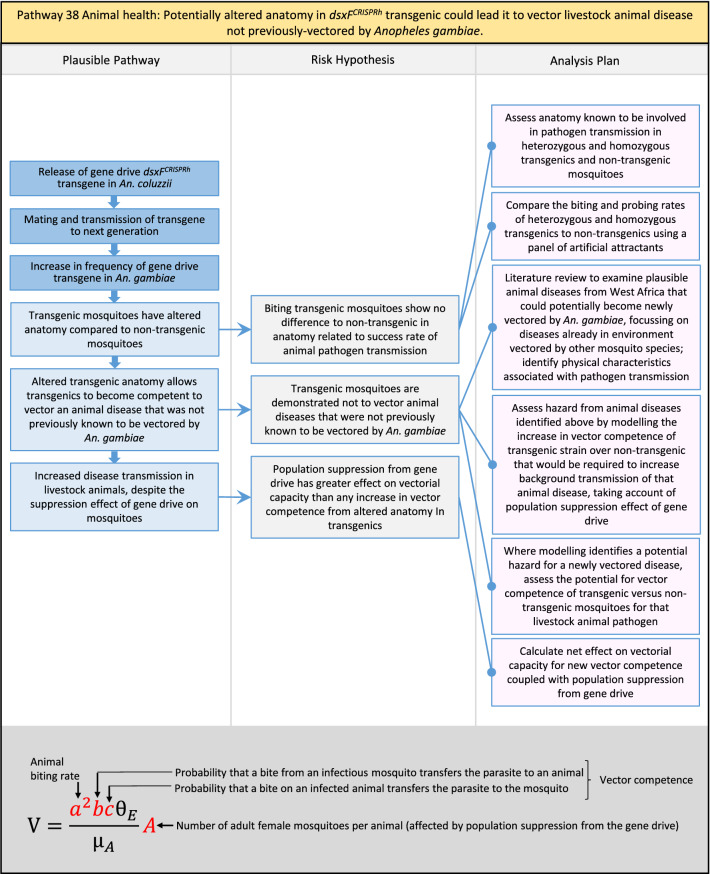
Pathway 38 Animal health: Potentially altered anatomy in *dsxF*^*CRISPRh*^ transgenic could lead it to vector livestock animal disease not previously-vectored by* Anopheles gambiae*. This pathway is about the efficiency of transmission, so any change in anatomical characteristics in the transgenics may increase the biting or probing rates or might increase the disease transmission rates. As *dsxF*^*CRISPRh*^ transgenic mosquitoes have reported anatomical alterations [17], anatomical structures implicated in disease transmission, such as the cibarial armature, can be examined in transgenics [102]. The net effect of a population suppression gene drive would ultimately reduce the impact of this specific harm by reducing the density of mosquitoes, including transgenic ones. The components of vectorial capacity (V) that would be affected in this pathway are shown in red in the equation

**Fig. 41:**
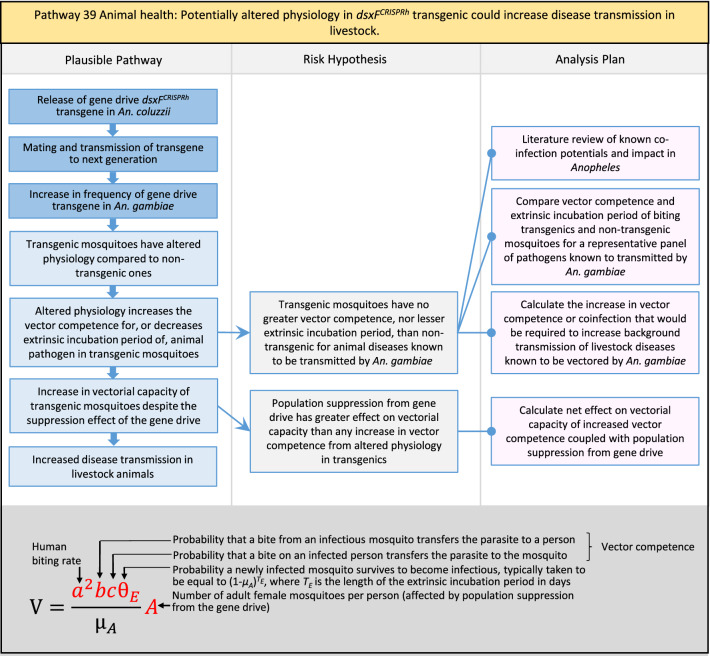
Pathway 39 Animal health: Potentially altered physiology in *dsxF*^*CRISPRh*^ transgenic could increase disease transmission in livestock. This pathway is about the efficiency of transmission, so any change in physiological characteristics in the transgenics, such as immune system function or capacity for co-infection or extrinsic incubation period [151], might increase the disease transmission rates of a given animal pathogen. The net effect of a population suppression gene drive would ultimately reduce the impact of this specific harm by reducing the density of mosquitoes, including transgenic ones. The components of vectorial capacity (V) that would be affected in this pathway are shown in red in the equation

**Fig. 42:**
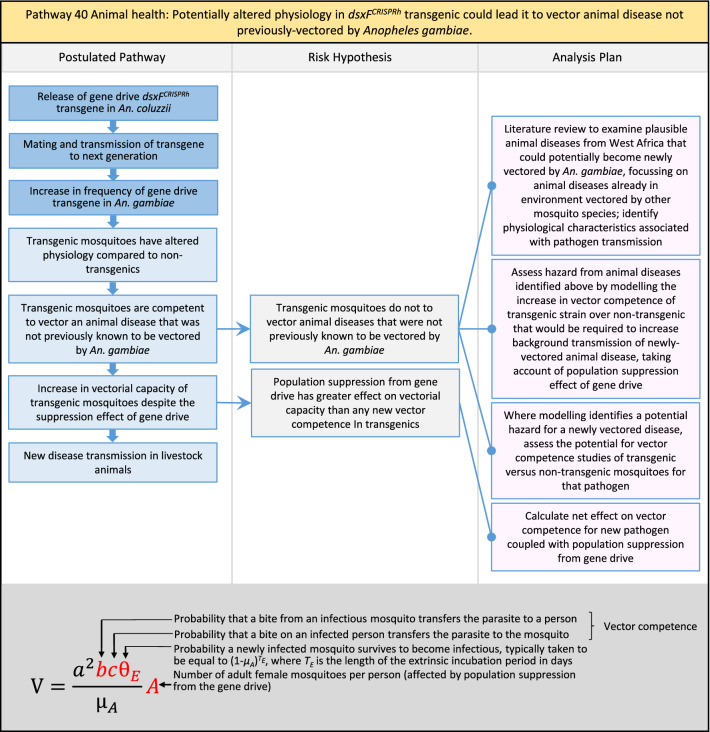
Pathway 40 Animal health: Potentially altered physiology in *dsxF*^*CRISPRh*^ transgenic could lead it to vector animal disease not previously vectored by* Anopheles gambiae*. Relevant physiology would include immune system function or capacity for co-infection. These newly transmitted diseases would already be present in the environment into which the transgenics were released. The net effect of a population suppression gene drive would ultimately reduce the impact of this specific harm by reducing the density of mosquitoes, including transgenic ones. The components of vectorial capacity (V) that would be affected in this pathway are shown in red in the equation

**Fig. 43:**
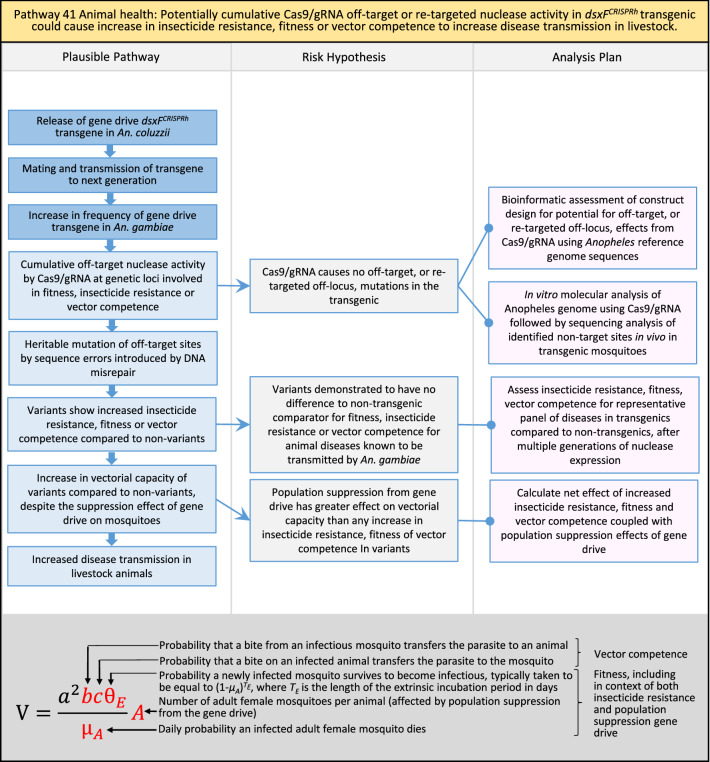
Pathway 41 Animal health: Potentially cumulative Cas9/gRNA off-target, or re-targeted, nuclease activity in *dsxF*^*CRISPRh*^ transgenic could cause increase in insecticide resistance, fitness or vector competence to increase disease transmission in livestock. The net effect of the population suppression gene drive should ultimately be to reduce this specific harm by reducing the density of mosquitoes including variants. For this potential harm, the first tier of the analysis would involve bioinformatic and molecular assessments of the potential for off-target or re-targeted mutations to occur in the transgenic. In the event of such mutations being detected, a second tier of phenotypic characterisations would then be performed. The components of vectorial capacity (V) that would be affected in this pathway are shown in red in the equation

**Fig. 44:**
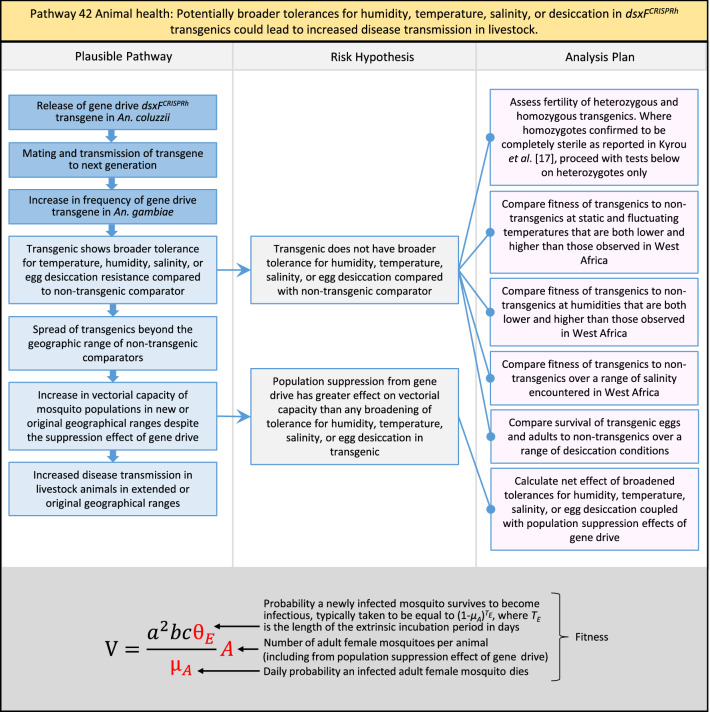
Pathway 42 Animal health: Potentially broader tolerances for humidity, temperature, salinity, or desiccation in *dsxF*^*CRISPRh*^ transgenics could lead to increased disease transmission in livestock. Were the transgenic to show a broadening of tolerance for environmental conditions, this could result in increased competition with existing species in its current geographic range, as well as new competition with new species in new range, in each case potentially increasing animal disease transmission. Transgenics with broadened tolerance for humidity and temperature might, for example, be expected to show extended survival into dry season compared to non-transgenic. The net effect of the population suppression gene drive should ultimately be to reduce this specific harm by reducing the density of mosquitoes including transgenic ones. The components of vectorial capacity (V) that would be affected in this pathway are shown in red in the equation

**Fig. 45:**
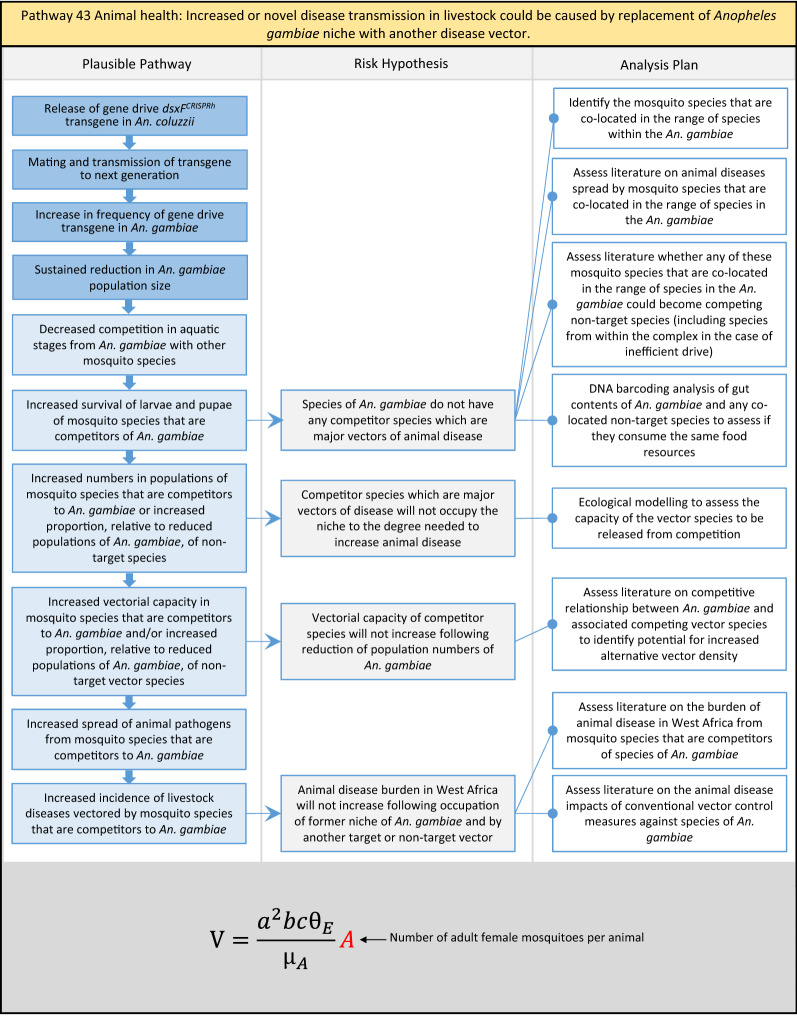
Pathway 43 Animal health: Increased or novel disease transmission in livestock could be caused by replacement of* Anopheles gambiae* niche with another disease vector. In this pathway, population suppression of *An. gambiae* could release other disease vectors from competition, leading to an increase the population density of those other disease vectors and therefore increase their vectorial capacity and disease transmission in livestock. The components of vectorial capacity (V) that would be affected in this pathway are shown in red in the equation

**Fig. 46:**
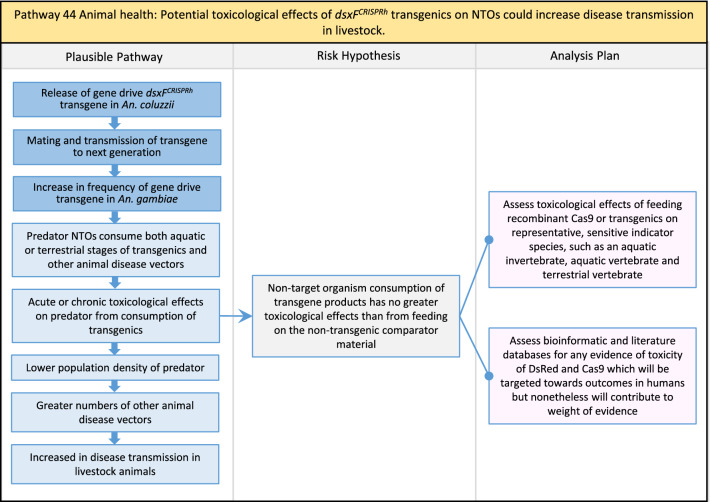
Pathway 44 Animal health: Potential toxicological effects of *dsxF*^*CRISPRh*^ transgenics on NTOs could increase disease transmission in livestock. Bioinformatic and literature evidence of any toxicity of DsRed and Cas9 in the analysis plan will likely be more targeted towards outcomes in humans but nonetheless will contribute to weight of evidence supporting or refuting this pathway. Defining the experimental conditions and choices of indicator species for chronic and acute studies will most likely involve discussion with national regulators, with reference to international regulatory guidance and best practice [26, 95]

**Fig. 47:**
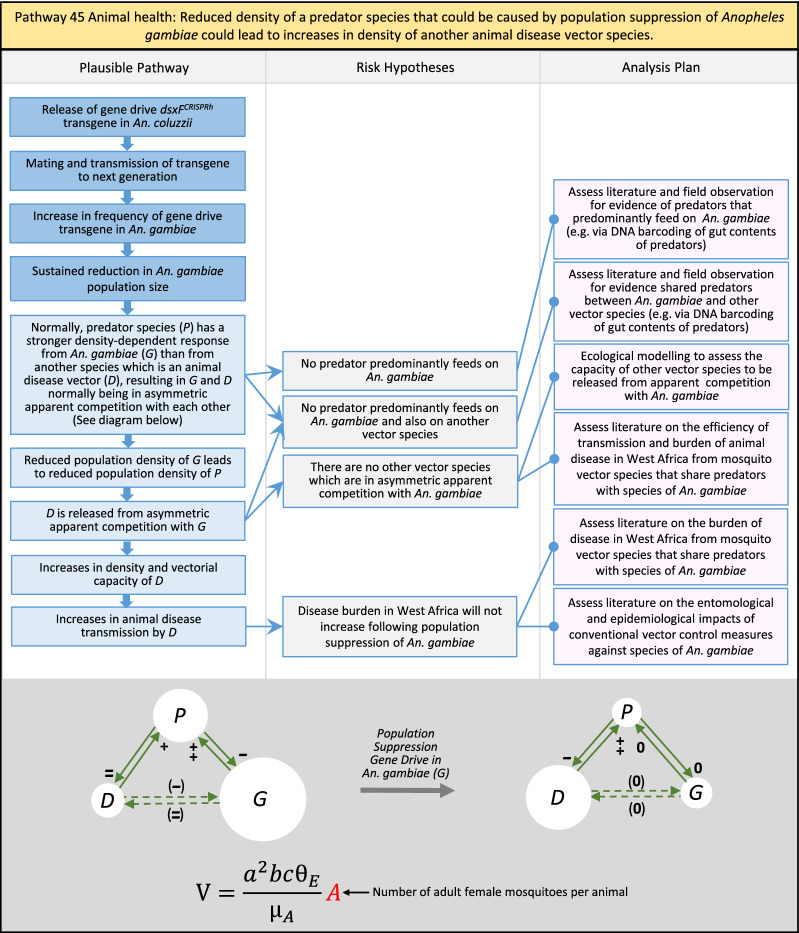
Pathway 45 Animal health: Reduced density of a predator species that could be caused by population suppression of* Anopheles gambiae* could lead to increases in density of another animal disease vector species. Species *P* denotes a predator of both *An. gambiae* (*G*) and an animal disease vector species, *D*. Solid green lines represent direct effects of one species on another. Dashed green lines indicate indirect effects from apparent competition [109]. Size of white circles denotes notional size of species populations. The + symbol denotes a positive effect on the species at the arrowhead, with + + indicating stronger positive effects. The − symbol denotes a negative effect on the species at the arrowhead, with − − indicating stronger negative effects. 0 denotes a negligible effect on the species at the arrowhead. When symbols are within parentheses, this denotes an indirect effect on the species at the arrowhead. In this pathway, the asymmetric apparent competition between *G* and *D* (for example, see Fig. 1b in [109]), before population suppression gene drive is introduced in *G*, is lost following population suppression gene drive introduction, leading to reductions in the density of *P* and increases in the density of *D*. Sustained reduction in the population of *An. gambiae* could also lead to reduction in the density of a valued predator if that predator would, for example, feed predominantly on *An. gambiae* in the wet season and then switch to feeding on, and controlling the numbers of, another disease vector or pest in the dry season, when *An. gambiae* would not typically act as its predominant food source during that period. The components of vectorial capacity (V) that would be affected in this pathway are shown in red in the equation

**Fig. 48:**
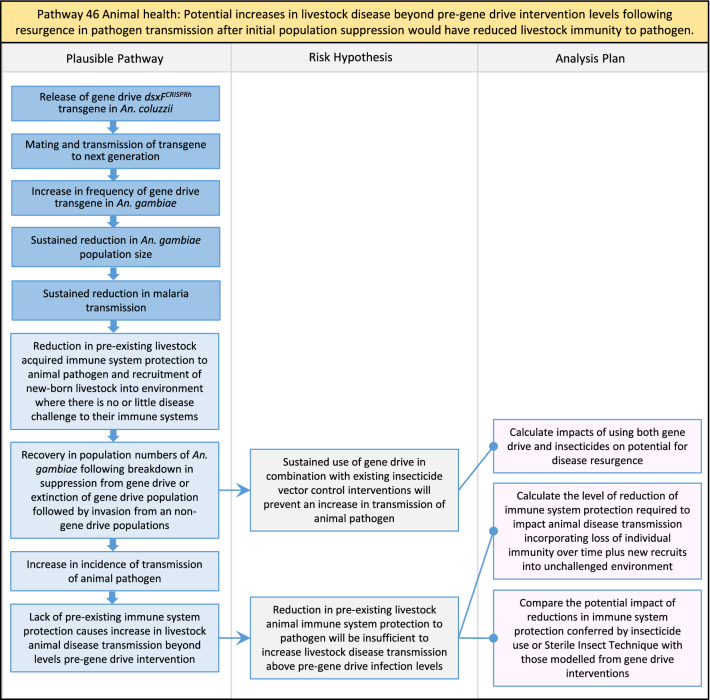
Pathway 46 Animal health: Potential increases in livestock disease beyond pre-gene drive intervention levels following resurgence in pathogen transmission after initial population suppression would have reduced livestock immunity to pathogen. This could a potential harm for any successful vector control agent, not just population suppression gene drive, but is included for the sake of completeness. The analyses here are likely to lead to a ‘weight of evidence’ in favour or against the potential harm [42], rather than a definitive conclusion

### Plausible pathways to potential harms to biodiversity protection goals

For biodiversity protection goals, eight plausible pathways to potential harm were identified, shown in Figs. 3, 4, 5, 6, 7, 8, 9 and 10. Conceptual models of three examples of these pathways are described. Potential harms involving wildlife were considered under biodiversity protection goals, whereas the health of livestock, domestic or companion animals were considered under animal health protection goals.

#### Pathway 1. Biodiversity: Potential toxicological effects of dsxF^*CRISPRh*^ transgenics on NTOs could reduce ecosystem services

Pathway 1 involves toxicological effects from consuming *dsxF*^*CRISPRh*^ transgenic material leading to a reduction in the density of predator, decomposer/scavenger or pollinator NTOs (Fig. 3). The plausible pathway to the potential harm illustrated how, following release and spread of the *dsxF*^*CRISPRh*^ transgene, predators could eat living transgenics, decomposers or scavengers could eat dead transgenic material, or pollinators could consume water from aquatic habitats containing dead transgenics and their breakdown products. Transgenic material could then cause direct acute or chronic toxicological effects on these NTOs, reducing their population densities in the ecosystem. This pathway could also be relevant to water quality, human health and animal health protection goals in an indirect way, for example, by increases in the densities of other pest or vector species, if the predator were to feed on both *An. gambiae* and those other species.

Two risk hypotheses were developed to interrogate steps of this pathway. One hypothesized that NTO consumption of *dsxF*^*CRISPRh*^ transgenic products would have no greater toxicological effects than from consumption of non-transgenic mosquito material. This could be tested in two ways. Firstly, bioinformatic and literature databases could be searched for any evidence of toxicity of Cas9 or DsRed. Such evidence would likely be more targeted towards toxicological outcomes in humans, but nonetheless could contribute to the weight of evidence corroborating or invalidating this risk hypothesis. Secondly, in toxicology studies recombinant transgenic proteins, such as Cas9, could be fed to representative sensitive indicator species. Defining the precise experimental conditions and choices of indicator species for these studies would most likely involve discussion with national regulators, with reference to international or other regulatory guidance and best practice, such as European Food Safety Authority’s guidance for the risk assessment of genetically modified animals and food and feed derived from them [26, 95].

A second risk hypothesis stated that levels of the transgene would be too low to produce toxic effects in NTOs. This could be tested by assessing the level of expression of transgenic products in the *dsxF*^*CRISPRh*^ gene drive strain and then extrapolating anticipated levels of transgenic biomass in aquatic or terrestrial habitats. Assuming the intended efficacy at most locations in the release area, as the population suppression from the gene drive takes effect, there should be decreasing densities of *An. gambiae* and, therefore, decreasing likelihood that NTOs would be exposed to any potential toxicological effects.

#### Pathway 3. Biodiversity: Potentially cumulative Cas9/gRNA off-target or retargeted nuclease activity in dsxF^*CRISPRh*^ transgenics could cause broader tolerances for humidity, temperature, salinity, or egg desiccation to reduce densities of valued species or ecosystem services

Pathway 3 involves the potential for off-target Cas9 nuclease activity [91], or retargeted off-locus nuclease activity [92], to generate mutations that could produce broader tolerances in *dsxF*^*CRISPRh*^ transgenics for a range of environmental conditions that would then allow them to spread beyond the geographic range of non-transgenic comparators (Fig. 5). The plausible pathway to this potential harm illustrates how, following release and spread of the *dsxF*^*CRISPRh*^ transgene, off-target or retargeted Cas9 nuclease activity could lead to misrepair and heritable mutations at genetic loci conferring broader phenotypic tolerances for environmental conditions such as temperature, humidity or salinity. This could then result in either increased competition from these variants with existing species within the current range of *An. gambiae* via extended survival into dry season compared to non-variants, or new competition with new species, or new populations of the same species, in new ranges.

As off-target mutations have been detected for a variety of guide RNAs in human cell lines ranging in frequencies from 0.03 to 60.1% [96], two manifestations of this pathway, in terms of ‘phenotypic changes due to mutation generation followed by natural selection’ *versus* ‘phenotypic changes due to mutation pressure’, were considered.

The former could be rare and difficult to observe reliably in the laboratory setting. Indeed, off-target single nucleotide variants have been detected in CRISPR-Cas9 edited mouse embryos at a frequency close to the spontaneous mutation rate [97], while no off-target mutations were detectable in some strains of transgenic mosquitoes [93]. Therefore, the impact from such off-target mutations would be on the same scale as spontaneous mutations that would in any event be occurring naturally in large field populations of wild-type mosquitoes [97].

However, the latter case, phenotypic changes due to mutation pressure, would represent a plausible pathway to potential harm from the intervention, as it would arise from higher mutation rates than spontaneous levels. Moreover, these should be observable under laboratory conditions. Therefore, four risk hypotheses were developed to test the validity of steps in this pathway supported by a tiered analysis plan [47, 80]. In the first tier, the hypothesis that Cas9 causes no off-target cutting in the *dsxF*^*CRISPRh*^ transgenic could be tested using bioinformatic assessments combined with *in vitro* experiments to determine if Cas9 would be predicted to cut non-target sequences, followed by genome sequencing in transgenics of any identified off-target sites to determine whether any of those sites would be the subject of off-targeting and mutation by misrepair *in vivo* and, if so, at what level.

A second tier of the analysis plan would test the hypothesis that the variants would have no broader tolerances for abiotic conditions outside the range of non-transgenic comparators. Here, measurement endpoints would involve assessment of the fitness of transgenics at lower and higher temperatures, humidity and salinity ranges than those found currently in West African for *An. gambiae*.

A third risk hypothesis posited that population suppression from gene drive would have a greater effect on population densities than any broadening of tolerance for humidity, temperature, salinity, or egg desiccation in variants and could be evaluated by calculating the net effect of identified changes in tolerances for environmental conditions and expected levels of population suppression. Assuming the intended efficacy at most locations in the release area (Fig. 2), the net effect of a population suppression gene drive could ultimately reduce this potential harm by reducing the density of mosquitoes, including variants.

A fourth risk hypothesis stated that *An. gambiae* did not have competitors that were either valued species or that provide essential ecosystem services which could be assessed via literature review.

#### Pathway 5. Biodiversity: Potential horizontal gene flow of the dsxF^*CRISPRh*^ transgene to a NTO eukaryote could lead to its unintended population suppression, thus reducing densities of valued species or ecosystem services

Pathway 5 identifies how the horizontal transfer of the *dsxF*^*CRISPRh*^ transgene could lead to the population suppression of an NTO eukaryote that is a valued species, thus affecting biodiversity (Fig. 7) [98]. The plausible pathway to this potential harm illustrates how, following release and spread of the *dsxF*^*CRISPRh*^ transgene, bacteria or viruses that are pathogenic to the valued species could acquire the transgene either directly, or via bacteriophage, or transposon. The virus or bacteria transformed with the *dsxF*^*CRISPRh*^ transgene could then infect the valued species leading to transfer of the transgene to its germline cells. A functional version of the transgene would then have to integrate into the guide RNA target site, with its regulated expression causing heritable transmission of the transgene and population suppression in the valued species.

Four risk hypotheses were developed to investigate this pathway. Firstly, it was hypothesized that *dsxF*^*CRISPRh*^ transgene would not get transferred from the transgenic to the germ cells of valued species on the expected timescale of a gene drive intervention, circa 10 years [99]. To test this hypothesis, the literature could be assessed to determine the likelihood and expected timescale required for a stretch of DNA of circa 7 kb, the size of the functional gene drive transgene, to be transferred from transgenics to valued species by non-sexual means, such as via transposon intermediaries. Hybridization was not considered a plausible mechanism for transfer of the gene drive transgene from *An. gambiae* to NTOs, including valued species. *Anopheles gambiae* is made up of *An. amharicus, An. arabiensis, An. bwambae, An. coluzzii, An. fontenillei, An. gambiae s.s., An. melas, An. merus*, and *An. quadriannulatus* [48–51] and the guide RNA target sequence of the *dsxF*^*CRISPRh*^ transgene is conserved in all of the above species examined [17]. Transfer of this transgene between any of these species via hybridization would thus likely lead to functional gene drive and population suppression in those species. Thus, all of the above species are considered target organisms. By contrast, the most closely related species to *An. gambiae* outside of the complex is *An. christyi*, which differs morphologically, and is genetically distinct, from *An. gambiae*, with both species being separated by circa 9 million years of evolution [72]. The absence of observed gene flow between both species supports the lack of any significant hybridization between these species, so that for even less closely related species of *Anopheles*, hybridization with *An. gambiae* is considered implausible.

The next two risk hypotheses were designed to interrogate the step in the pathway whereby the transgene would integrate into the guide RNA target of the genome of germline cells in the valued species, a prerequisite for the transgene to result in gene drive. There could be two possibilities: the first was represented by the risk hypothesis that the transgene would not insert randomly into the genome at the target site and could be tested by calculating the probability that the transgene (circa 7 kb) would insert at the guide RNA target site (23 bp) of the genome of a valued species’ germline cells. For reference, the *An. gambiae s.s.* genome is some 278 Mb [100]. The second possibility was represented by the risk hypothesis that the Cas9 and guide RNA of the transgene would not be expressed, including transiently, so that the transgene could integrate faithfully into the target site. This would be assessed via literature review of the functionality of regulatory and coding sequences of the transgene in the germline of valued species of insects.

A fourth risk hypothesis postulated that the guide RNA target site would not be conserved in the genomes of valued species. Bioinformatics could be used to assess the degree of conservation of the RNA target site in valued species of insects associated with *An. gambiae*.

### Plausible pathways to potential harms to water quality protection goals

For water quality protection goals, two plausible pathways to potential harm were identified (Figs. 11 and 12). The conceptual model for pathway 9 is described below.

#### Pathway 9. Water quality: Potential adverse impact on quality of water, and its flora and fauna, from reduced nutrient composition of aquatic habitats could be caused by potential toxicity of dsxF^*CRISPRh*^ transgenic products

For Pathway 9, the accumulation of transgenic product could lead to toxicity in detritivores, leading to negative effects on water quality for aquatic NTO flora and fauna (Fig. 11). Four risk hypotheses were developed to interrogate this pathway. The first posited that the population suppression gene drive would inhibit the accumulation of transgenic products by decreasing the density of aquatic stages of the TO. This could be tested by calculating the net effects of accumulation of transgenic products from mortality rates in aquatic stages of transgenics and reduced density of aquatic transgenics via population suppression. The second risk hypothesis stated that the transgenic products, or indeed their amino acid breakdown products, would not accumulate in water. This could be tested in the analysis plan by assessing the literature for evidence on the environmental fate of transgenic and breakdown products, informed by quantitative analysis of their levels of expression in transgenics. A third risk hypothesis posited that transgenic products would not accumulate in sediment, which could be tested by both literature and assessing the environmental fate of transgenic products in sediments. The fourth risk hypothesis stated that transgenic products were not toxic. This could be tested in the analysis plan by bioinformatic and literature assessments for any evidence of toxicity of transgenic products in animals, as well as assessing the data from NTO toxicology studies described for Pathway 1 above.

### Plausible pathways to potential harms to human health protection goals

For human health protection goals, 20 plausible pathways to potential harm were identified (Figs. 13, 14, 15, 16, 17, 18, 19, 20, 21, 22, 23, 24, 25, 26, 27, 28, 29, 30, 31, 32). Conceptual models for four examples of such pathways are described below. In many but not all cases, plausible pathways to potential harm for human health were mirrored in pathways for animal health.

#### Pathway 11. Human health: Transgenic proteins could cause specific allergic or toxicological responses in humans from dsxF^*CRISPRh*^ transgenic bites beyond responses to non-transgenic bites

Pathway 11 describes how expression of transgenic proteins DsRed or Cas9 in the saliva of *dsxF*^*CRISPRh*^ transgenic mosquitoes could lead to allergic responses in humans from transgenic biting beyond those that occur from non-transgenic biting (Fig. 13). This pathway was based on the potential harm that could be caused to an individual human from exposure to transgenic proteins. It could, therefore, occur in the first generation after release of the transgenics, or at subsequent stages, for example, when the transgene increases in frequency in the mosquito population (Fig. 2).

The first risk hypothesis to investigate this pathway stated that Cas9 or DsRed were not detectable in the saliva of transgenic mosquitoes. The measurement endpoint to test this hypothesis relies on tiered assessment. Firstly, it would be determined whether Cas9 or DsRed were expressed in salivary glands in heterozygous *dsxF*^*CRISPRh*^ transgenic mosquitoes that, unlike homozygotes, can bite. If those transgenic proteins were present in salivary glands, it would next be determined whether Cas9 or DsRed were present in the saliva of the transgenic mosquitoes. A second risk hypothesis proposed that the Cas9 and DsRed transgenic proteins did not cause increased allergenicity or toxicity in humans. This could be assessed by searches of bioinformatic and literature databases to determine if there was evidence of allergenicity of Cas9 or DsRed and by assessing toxicology data from selected NTO studies as outlined in the Analysis Plan of Pathway 1.

#### Pathway 21. Human health: Potentially altered anatomy, or host-seeking behaviour, in dsxF^*CRISPRh*^ transgenics could increase the transmission of human diseases, including lymphatic filariasis

Pathway 21 relates to how altered anatomy in transgenics could lead to increases in the transmission of lymphatic filariasis (LF) (Fig. 23). After malaria, LF is the most burdensome disease transmitted by species of *An. gambiae* in western sub-Saharan Africa. For example, in Burkina Faso, malaria accounts for 17.96% of total disability-adjusted life years (DALYs), compared to 0.29, 0.05 and 0.0094% of total DALYs for LF, yellow fever and dengue, respectively [101]. Despite this, direct laboratory assessment of the vector competence of *An. gambiae* for the most common LF parasite, *Wuchereria bancrofti*, has not yet reliably been established [102].

Homozygous *dsxF*^*CRISPRh*^ transgenic mosquitoes have reported anatomical alterations, including specifically to mouthparts, with most heterozygotes appearing to be normal in this respect [17]. This pathway is about the efficiency of transmission, so any relevant change in anatomical characteristics in *dsxF*^*CRISPRh*^ transgenics may increase the probing rate of homozygous transgenics, or in heterozygotes increase the biting rate or the transmission rate from a given biting rate. Moreover, the cibarial armature is implicated in transmission of filariae to mosquitoes [103–105].

Four risk hypotheses were developed to investigate this pathway. In the first, it was hypothesized that transgenics did not differ anatomically to non-transgenics in anatomy related to the success of pathogen transmission. This could be assessed in transgenics and non-transgenics in insectary anatomical studies. The second risk hypothesis posited that transgenics did not bite humans more than non-transgenics to increase disease transmission. This could be tested in the laboratory by comparing the biting and probing rate of transgenics with non-transgenics using a panel of artificial attractants, and the increase in the number of infectious bites required to increase background disease transmission could be calculated. A third risk hypothesis proposed that transgenics showed no increase in vector competence, or decrease in extrinsic incubation period, compared with non-transgenics. Both of these parameters could be assessed in the laboratory for a representative panel of pathogens vectored by *An. gambiae*. A fourth risk hypothesis proposed that population suppression from gene drive would have a greater effect on vectorial capacity than any increase in vector competence caused by altered *dsxF*^*CRISPRh*^ transgenic anatomy. This hypothesis could be tested by calculating the net effect on vectorial capacity of any such increase in vector competence in the presence of population suppression from gene drive.

#### Pathway 25. Human health: Potentially cumulative Cas9/gRNA off-target or retargeted nuclease activity in dsxF^*CRISPRh*^ transgenics could cause heritable increase in insecticide resistance, fitness or vector competence to increase human disease

Pathway 25 describes how the accumulation in transgenics of Cas9/guide RNA off-target or retargeted mutations in genomic loci involved in insecticide resistance, fitness or vector competence could lead to an increase in vectorial capacity and human disease transmission (Fig. 27). Three risk hypotheses were constructed for this pathway, supported by a tiered analysis plan [47, 80]. In the first, Cas9/guide RNA was hypothesized to cause no off-target or retargeted mutations in transgenics, which could be tested firstly by bioinformatic assessment on the potential for off-target or retargeted effects using *Anopheles* 1,000 genomes reference genome sequence [106, 107], and secondly by genomic sequence analysis (e.g. using *in vitro* CIRCLE-Seq [108]) of mosquito DNA to identify potential non-target sites. These non-target sites could then be sequenced in transgenics to search for *in vivo* evidence of their mutation [93].

Where off-target or retargeted mutations would be detected in transgenics by molecular and bioinformatic analyses, as discussed for Pathway 3 above, they could occur via two scenarios: ‘phenotypic changes due to mutation generation followed by natural selection’ or ‘phenotypic changes due to mutation pressure’. The former may not be observable in the laboratory but would, in any event, be occurring naturally in large field populations in the absence of the transgene [34, 93, 97]. The latter does however represent a plausible pathway to potential harm caused by this transgene but would require high mutation rates that should be observable under laboratory conditions [93]. Therefore, to address this latter scenario, a second tier of the analysis plan would be to test a second risk hypothesis that the off-target or retargeted variants show no difference in vectorial capacity, which could be tested experimentally by comparing the insecticide resistance, fitness and vector competence for a representative panel of diseases in transgenics with non-transgenics following multiple generations of nuclease expression in the germline.

A third hypothesis proffered that population suppression from the gene drive would have a greater effect on vectorial capacity than from any increase in insecticide resistance, fitness or vector competence, the net effects of which could be calculated empirically.

#### Pathway 29. Human health: Potentially reduced density of a predator species caused by population suppression of *Anopheles gambiae* could lead to increases in density of another human disease vector species

Pathway 29 describes how population suppression of *An. gambiae* could cause reductions in the density of a predator that leads to and expansion in the density of another human disease vector (Fig. 31). This would occur because, under normal circumstances, the predator species *P* would have stronger density-dependent response from feeding on *An. gambiae* (*G*) than from feeding on another species (*D*) which is a human disease vector, so that under such normal circumstances *G* and *D* would be in asymmetric apparent competition with each other [109]. However, with population suppression gene drive in *An. gambiae*, the population density of *P* could also reduce. This could, in effect, release *D* from asymmetric apparent competition with *G*, leading to increases in the population density and vectorial capacity of *D*, resulting in increased disease transmission in human by *D*.

Five risk hypotheses were developed to interrogate this pathway. In the first, it was posited that no predator predominantly feeds on *An. gambiae*. This could be tested in the first tier of an analysis plan by assessing the literature and field observations for evidence of predators that predominately feed on *An. gambiae*, such as from studies DNA barcoding the gut contents of predators [110]. A second risk hypothesis, which stated that no predator that predominately feeds on *An. gambiae* also feeds on another human disease vector species, would only need to be investigated where the first risk hypothesis was falsified. This could be tested in the second tier of the analysis plan by further literature investigation and field observations for evidence of shared predators between *An. gambiae* and other vector species.

A third risk hypothesis posited that there are no other human disease vectors in asymmetric apparent competition with *An. gambiae*, which could be investigated via ecological modelling. A fourth risk hypothesis proffered that *D* is a less efficient vector of diseases that are also transmitted by *An. gambiae* and/or the diseases transmitted by *An. gambiae* are more harmful than those transmitted by *D.* This could be tested by modelling the net effect of disease transmission of *An. gambiae* and *D* both before and after population suppression of *An. gambiae*, as well as assessing the literature on the burden of human disease in West Africa from mosquito species that share predators with *An. gambiae*.

The fifth, and last, risk hypothesis for this pathway stated that the disease burden in West Africa will not increase following population suppression of *An. gambiae*, which would be tested by assessing the literature on the entomological and epidemiological impacts of conventional vector control measures against *An. gambiae*, such as indoor residual spraying or insecticide-treated bed nets [111].

### Plausible pathways to potential harm for animal health protection goals

For animal health protection goals, 16 plausible pathways to potential harm were identified (Figs. 33, 34, 35, 36, 37, 38, 39, 40, 41, 42, 43, 44, 45, 46, 47, 48). Conceptual models for two of these pathways are described below. In most cases, the animals considered were livestock, but these pathways could equally be applicable to domestic or companion animals. Potential harms to wildlife were considered under biodiversity protection goals. In all cases, pathways to potential harm for animal health were mirrored in pathways for human health, so that the examples presented below are also relevant to plausible pathways to potential harm for human health protection goals.

#### Pathway 32: Animal health: Potentially decreased mosquito defence response to pathogen in dsxF^*CRISPRh*^ transgenics from altered levels of endogenous RNA, protein or microbiome could lead to increased disease in livestock

Pathway 32 defines how decreases in transgenic mosquito defence responses via alterations to endogenous RNA, protein or microbiomic species could lead to increased livestock disease (Fig. 34). Mosquito RNA or protein in saliva, midgut or haemolymph, or contents of the microbiome, can alter defence responses to human pathogens such as *Plasmodium* or O’nyong’nyong virus (ONNV) [112–116]. It was, therefore, considered to be plausible to envisage that the same process could be modulating mosquito responses to animal pathogens. Any such alterations in transgenics compared to non-transgenics could alter the vector competence of transgenics and thus vectorial capacity.

Two risk hypotheses were developed to interrogate this pathway. In the first, it was proposed that transgenics would not transmit higher levels of animal pathogens than non-transgenics, which could be tested in the analysis plan using measurement endpoints in vector competence assays involving a representative panel of pathogens. The second hypothesis stated that population suppression from gene drive would have a greater overall negative effect on livestock disease incidence than any increased levels of vector competence, the net effect of which could be calculated from empirical data.

#### Pathway 43. Animal health: Increased or novel disease transmission in livestock could be caused by replacement of* Anopheles gambiae* niche with another disease vector

Pathway 43 describes how population suppression of *An. gambiae* could release another animal disease vector species from competition, thereby increasing the incidence of livestock disease (Fig. 45). The pathway was considered to be most likely to manifest itself in aquatic habitats of larval development, which have the most impact on vectorial capacity of *An. gambiae* [117, 118]. Diminishing density of larvae or pupae of *An. gambiae* could cause decreased competition with other mosquito vector species. This would then cause increases in the vectorial capacity of those competitor vectors, increasing the spread of animal pathogens in livestock.

Four risk hypotheses were developed to investigate steps in this pathway. In the first, it was hypothesized that species of *An. gambiae* do not have competitor species that are major vectors of animal disease. This could be tested by identifying vector species that share aquatic habitats with species of *An. gambiae*, assessing animal diseases spread by those species, examining published evidence for competition between these species and *An. gambiae* and DNA barcoding analysis of gut contents of *An. gambiae* and any co-located non-target species to assess if they consume the same food resources [110]. A second hypothesis was developed which stated that competitor species which are major vectors for disease would not occupy the *An. gambiae* niche to the degree needed to increase animal disease, which could be assessed in the analysis plan by ecological modelling. A third risk hypothesis stated that the vectorial capacity of competitor species would not increase following reduction in the density of *An. gambiae* and could be tested by assessing literature on the competitive relationship between *An. gambiae* and associated competing vector species to identify potential for increased alternative vector density. The fourth risk hypothesis posited that the animal disease burden in West Africa would not increase following occupation of the former niche of *An. gambiae* by another target or non-target vector species. As with the analysis plan in Pathway 27, this could be tested by assessment of the literature on the burden of animal disease in West Africa from mosquito species that are competitors of *An. gambiae* and on the impacts on animal disease from conventional malaria vector control measures, such as the use of insecticides, against *An. gambiae*.

## Discussion

Drawing on protection goals identified from previous, published consultative exercises on gene drive involving a broad base of expertise from across Africa and under the direction of AUDA-NEPAD [45, 46], problem formulation was used here as the initial step in an ERA of the hypothetical release of the *dsxF*^*CRISPRh*^ transgene-based population suppression gene drive for malaria vector control in West Africa. Eight potential adverse effects to protection goals were identified, such as reduced density of valued species or ecosystem services, or increased disease transmission in humans. In total, 46 plausible pathways to potential harms to protection goals to health and the environment were developed (Table 2). Each pathway was categorized by protection goal, cause, effect, correlation of exposure levels with transgene efficacy, and relevance to ERAs of other transgenic mosquito strains (Table 2). Although some of the pathways identified here were based on considerations arising from specific anatomical alterations observed in homozygous *dsxF*^*CRISPRh*^ transgenics (n = 14), many of these could still be applicable to field releases of other strains of transgenic mosquito (n = 18). Other pathways could be selectively applicable to population suppression gene drive strains (n = 8), CRISPR-Cas9-based strains (n = 3) or other gene drive transgenic strains (n = 2) (Table 2).

### Causes of potential harms

The intended outcome of population suppression gene drive in *An. gambiae*, decreased density of the target species, was not itself considered to be a potential harm, given that there is no evidence to support the complex playing a key role in ecosystems or provision of ecosystem services [75]. Indeed, population suppression of these TOs is by definition an accepted outcome for any existing malaria vector control programme.

Most causes of harm related to changes in the biology of transgenics compared to non-transgenics at the level of the individual mosquito. For example, homozygous *dsxF*^*CRISPRh*^ transgenic females are sterile, whereas heterozygotes are fertile [17]. Homozygous *dsxF*^*CRISPRh*^ transgenic females also have anatomical defects in the proboscis so that they cannot bite. Homozygous *dsxF*^*CRISPRh*^ transgenic females also show additional anatomical alterations in the antennae, male accessory glands, spermatheca, claspers, cercus and pupal genital lobe [17]. Given the presence of distinct visible anatomical defects in homozygous *dsxF*^*CRISPRh*^ transgenics, as yet unobserved changes to physiology or behaviour in homozygous transgenic females were also considered plausible during the development of these pathways. Indeed, altered anatomy, physiology or behaviour in individual *dsxF*^*CRISPRh*^ transgenics represented the most common category of cause of potential harm (n = 24), with sub-sets of this causes of potential harm category including changes in transgenic vector competence (n = 14) or in host biting rates (n = 5).

The second-most common category of causes of potential harm was fitness effects in transgenics (n = 11), with either increases in transgenic fitness (n = 8) or fitness costs in individuals possessing the *dsxF*^*CRISPRh*^ transgene (n = 3). Increases in fitness could, for example, allow transgenics to extend their geographic range or contribute directly to increases in vectorial capacity unless the efficacy of population suppression were to outweigh the effect. Fitness costs in the transgenic were also causes of potential harms to water quality from increased larval and pupal mortality (n = 2). After fitness effects, the next most common category of cause of harm was from the transgenic containing a toxin or allergen (n = 9).

As well as potential harms caused by changes to the biology of transgenic mosquitoes at the individual level, some potential harms were identified that would occur at population level as a result of a population suppression gene drive (n = 7). Unlike those potential harms reported in Roberts et al. [45] and Teem et al. [46], plausible pathways to potential harm were developed which would be caused by horizontal gene transfer of the population suppression gene drive transgene cassette (n = 2), potentially leading to population suppression of a valued species of NTO (n = 1). As described in Methods, vertical gene transfer of the transgene via hybridization amongst species of the *An. gambiae* complex was considered to be an intended effect and not a harm itself, because spread of the gene drive transgene to all species of the *An. gambiae* complex is an expected outcome.

In other plausible pathways to potential harm, effective population suppression of *An. gambiae* would, of itself, be the primary cause of potential harm by leading to the release of other species from competition (n = 3; Pathways 7, 27, 43 in Figs. 9, 29, 45, respectively) or apparent competition (n = 2; Pathways 29 and 45 in Figs. 31 and 47, respectively) or the eventual loss of acquired or herd immunity to diseases transmitted by *An. gambiae* (n = 2; Pathways 30 and 46 in Figs. 32 and 48, respectively). It is worth noting, however, that any successful vector control programme has the potential to cause such consequent harms. For example, between 1955 and 1959, house residual spraying vector control programmes using dieldrin in East Africa resulted in almost complete elimination of malaria transmission, which correlated with decreases in the population densities of *Anopheles funestus* and concomitant sharp increases in densities of either *Anopheles rivulorum* or *Anopheles parensis*, which were attributed to release of the latter species from competition with *An. funestus* in aquatic habitats [119–121]. In an example supporting the loss of acquired immunity following a conventional malaria control programme, a longitudinal study in Senegal between 2007 and 2010 tracked the impact of insecticide-treated bed nets and artemisinin-based combination therapy and found that the incidence of malaria initially decreased, but increased again around 30 months after the distribution of bed nets [122, 123]. This resurgence in malaria was most pronounced in adults and children 10 years of age or older, rising from 33 % to 2007 to 63 % in 2010, which was attributed, at least in part, to reduced levels of acquired immunity [122, 123], consistent with observations from other studies in Kenya, The Gambia and Uganda [124–127].

One direct cause of potential harm arising from *dsxF*^*CRISPRh*^ transgenics is worth some discussion, as it would not occur for non-nuclease expressing transgenic mosquitoes that have already progressed to field releases [20]. Because gene drive is propagated by a DNA endonuclease expressed in *dsxF*^*CRISPRh*^ transgenics, it creates the possibility for the occurrence of promiscuous off-target or retargeted mutations [91], which would then subsequently lead to potential phenotypes in transgenics that might cause harms to protection goals, such as increased vector competence for diseases (see Pathways 3, 25 and 41 in Figs. 5, 27 and 43, respectively). The Cas9 enzyme is highly specific and the gRNA can be designed using reference *Anopheles* genomes to prevent sequences identical to the target site at genomic locations other than the target site. Guide RNAs can also be designed to limit the potential for off-target or re-targeted effects. However, unintended nuclease-induced mutation could theoretically become common in two ways: the mutation might happen to increase the fitness of the mosquito and be positively selected for, or off-target cleavage might be at such a high rate that it leads to a correspondingly high frequency of mutation in the population. The former simply reflects the *status quo* in large field populations where the full range of possible mutations are naturally occurring and potentially being selected for. The latter would require high mutation rates that would be observable in the laboratory. Determining the propensity for off-target or re-targeted mutations in gene drive transgenics early in the development of a strain for field release is, therefore, yet another essential aspect of the ERA for population suppression gene drive.

Mechanisms by which the efficacy of population suppression gene drive could be lost were also considered (Fig. 1). In particular, the evolution of resistance to gene drive, for example via the emergence of second-site suppressors, could also lead to pleiotropic effects that are in themselves potential harms, such as increase increased insecticide resistance (Pathway 18 in Fig. 20), vector competence (Pathway 20 in Fig. 22) or environmental tolerance (Pathway 26 in Fig. 28). This would be analogous to the situation where mutations conferring insecticide resistance to *An. gambiae* can have pleiotropic effects on vectorial capacity for *Plasmodium* [128–131]. Nonetheless, insecticides remain a cornerstone of malaria vector control programmes, despite significant remaining uncertainties on physiological interactions between insecticide resistance and vector competence and their potential contributions to disease transmission [132–135].

### Effects of potential harms

The effect of each potential harm was categorized as either direct (n = 30) or indirect (n = 16) (see Table 1). Pathways were also classified into eight broad categories of potentially harmful effects on protection goals. The largest two categories involved 26 pathways related to increases in disease transmission in humans (n = 13) or livestock (n = 13). The second largest category was reduced populations of valued species or ecosystem services (n = 8). The third largest category of potentially harmful effects involved increased toxicological effects and immune or allergic responses in humans (n = 5). Four other categories of potential harmful effects consisted of reduced water quality for humans, livestock or NTOs (n = 2), novel disease transmission in humans (n = 2) or livestock (n = 2), and increased toxicity in livestock (n = 1).

### Exposure routes to potential harms

The most common exposure route in pathways was from biting (n = 26), in which cases *dsxF*^*CRISPRh*^ heterozygous transgenics, but not homozygous transgenics, would provide the source of exposure. The potential for increased allergenicity in humans caused from exposure to transgenic mosquitoes via incidental inhalation or ingestion (Pathway 12 in Fig. 14; cf. [45]), which could occur from exposure to both homozygotes and heterozygotes, was also considered as plausible. The second most common exposure route to potential harms was from NTO consumption of the transgenic or changes in NTO consumption patterns caused by exposure to the transgenic (n = 6). The third most common exposure route was changes in predator-prey interactions (n = 3). Bio-accumulation of transgenic products or detritus and changes in competition were also routes of exposure for three potential harms each. Horizontal gene transfer, spread of transgenics beyond their geographic range and loss of immune system pre-exposure to pathogens were routes of exposure for two potential harms each.

### Exposure levels leading to potential harms

The relationship between level of exposure leading to potential harms and success of the intervention is worth considering, as this will guide future considerations of the risks and benefits from environmental releases of *dsxF*^*CRISPRh*^ transgenics. A minority of pathways (n = 4) could occur via allergic or immune system responses in individual humans exposed to potentially single transgenic mosquitoes or their body parts, and these could occur without any gene drive efficacy. Only 11 potential harms would be unrelated to the presence of the transgene in the environment and three, caused by off-target or retargeted mutations, could occur either due to the continued presence or subsequent absence of the transgene. However, for most potential harms to occur (n = 42), they would depend of the successful efficacy of the gene drive (Table 2).

The exposure level leading to the majority of potential harms (n = 35) would also be dependent on the absolute numbers of transgenics in the environment, where the greater the efficacy of population suppression, the less exposure there should be leading to potential harms. However, exposure levels leading to a minority of potential harms (n = 8) could increase with successful population suppression gene drive, such as in Pathways 6, 7, 27, 29, 30, 43, 45, and 46 shown in Figs. 8, 9, 29, 30, 43, 45, 47, and 48, respectively. These pathways could theoretically occur with any effective vector control tool that was relatively enduring and species specific, including pre-existing programmes based on insecticides or sterile insect technique (SIT) [122, 136]. In that sense, they represent plausible pathways to potential harm from successful malaria vector control, rather than from population suppression gene drive *per se*.

It is also important to recognize that any potential investigational release of a gene drive is first and foremost into a local environment with specific conditions which will vary from place to place and season to season [19]. So it can also be expected that the performance of the gene drive, and its suppression of the population, will vary temporally and spatially. There may also be oscillations in effects from the gene drive, for example reflecting the impact of the dry season on the survival of transgenics in highly seasonal locations (Fig. 2). This also means that exposure to any potential harm caused by the gene drive may also vary temporally and spatially [44].

### Risk hypotheses and experiments with widest impacts

Analysis of risk hypotheses and allied analysis plans revealed the experimental outputs that should provide the widest impact on testing of pathways and potential harms with the least levels of residual uncertainty. The most common theme amongst all risk hypotheses (in n = 14 pathways: 14, 20–25, 32, 36–41), that the transgenic would not transmit pathogens better than non-transgenics, can be informed by testing in the laboratory by assessments of vector competence and extrinsic incubation periods for a number specific representative pathogens. Data from such experimental evaluations can then further evaluated in the context of additional parameters relevant to vectorial capacity, to inform risk conclusions in subsequent stages of ERA. The second most common theme amongst risk hypotheses was that the transgenic was not toxic or allergenic (in n = 10 pathways: 1, 9, 11–13, 16, 17, 28, 31, 44), which can be tested initially from analysing bioinformatic and literature databases, supplemented by feeding studies in indicator species. The third most frequent theme in risk hypotheses involved fitness being unaffected in transgenics (in n = 10 pathways: 2, 3, 8, 10, 18, 25, 26, 34, 41, 42), which can be tested in small and large cage laboratory studies. The next most common theme in risk hypotheses was that transgenics did not bite more than non-transgenics (in n = 6 pathways: 19, 21, 22, 35, 37, 38). Rates of biting and probing can be assessed in the laboratory using a panel of artificial attractants. Finally, another common theme in risk hypotheses was that transgenic saliva did not differ from non-transgenic saliva (in n = 6 pathways: 11, 13, 15, 16, 31, 33), which can be tested via a variety of laboratory experiments that will assess levels of guide RNA, endogenous allergens, or RNA and proteins implicated in host defence responses.

### Dismissed pathways to potential harm

The exercise which led to the development of the current set of plausible pathways to potential harm took place between July 2019 and October 2020. Over that period, several provisional pathways were developed that were ultimately excluded from this analysis as invalid or inconsequential [34]. These included:

- n = 5 pathways to *loss of efficacy* of population suppression gene drive from the suite of initial pathways considered, as it was agreed they did not directly result in potential harms to protection goals and therefore should not be included amongst the plausible pathways to potential harm;
- n = 3 pathways related to *transboundary movement* as it was subsequently recognized that ERA does not formally include socio-economic or legal issues, such as the potential for transboundary movement of transgenics, despite these warranting further exploration in the context of gene drive organisms that are anticipated to cross national borders;
- n = 1 pathway to potential harm that would be caused by *vertical gene transfer* (via hybridization) of the population suppression gene drive from *An. gambiae* to species outside the complex, as it was subsequently recognized that the most closely related species to those within the *An. gambiae* complex is *An. christyi*, but which is separated by circa 9 million years of evolution. The absence of observed gene flow between species of *An. gambiae* and *An. christyi* supports the lack of any significant hybridization between these species so that, for even less closely related species of *Anopheles*, hybridization is considered implausible. Moreover, in species of *Anopheles* more distantly related to *An. gambiae* than *An. christyi*, the guide RNA target DNA sequence of the *dsxF*^*CRISPRh*^ transgene diverges from that found in *An. gambiae*;
- n = 2 pathways leading to potential increases in disease transmission caused by *increased transgenic larval mortality* because it was based on the false assumption that population suppression would automatically lead to increased larval mortality and because it was ultimately considered biologically implausible as a potential route to increased disease transmission;
- n = 6 pathways to potential harm to either human or animal health that would result in *transmission of a novel pathogen* caused by altered host-seeking behaviour in, or off-target mutations in, or extension in geographic range of, the transgenic. By contrast, pathways involving altered transgenic anatomy or physiology leading to the transmission of a novel pathogen were retained as plausible;
- n = 6 pathways, which were eliminated when n = 12 pathways were combined together into n = 6 pathways at the request of peer reviewers to help identify more efficiently common risk hypotheses and key experiments that could interrogate the viability of a number of pathways to a low level of remaining uncertainty.

## Limitations of this problem formulation exercise

The aim of this problem formulation exercise was to generate a comprehensive, if not exhaustive, set of plausible pathways to potential harm to previously defined protection goals from simulated field releases of the *dsxF*^*CRISPRh*^ transgene in West Africa. Although based on the gene drive strain containing the *dsxF*^*CRISPRh*^ transgene as described in Kyrou et al. [17], this work should also broadly be applicable to other similar CRISPR/Cas9-based population suppression gene drive strains targeting *doublesex*, for example those exploiting multiplexed guide RNAs (see Table 2). However, some of the pathways presented here may need to be re-examined on a case-by-case basis, depending on the strain under investigation and release protocol to be used. Indeed, new or altered pathways may also be identified as the scientific evidence base expands. These pathways are, therefore, likely to be revised and updated periodically, including with feedback from stakeholders and the wider scientific community.

Although the logical, stepwise process allows for the systematic identification of plausible pathways to potential harm, the strict linear format does not allow for branching pathways, parallel pathways or feedback loops. This may sometimes force the ‘shoehorning’ of parallel processes into a series of sequential events. The approach does, however, offer a transparent communication tool for the unbiased identification and description of pathways, potential harms and analysis plans.

Another difficulty encountered in this exercise was in defining the criteria for plausibility; pathways were considered plausible based on scientific and technical knowledge, but also based on expert judgement, introducing an element of subjectivity to the exercise [41]. This situation is not unique to problem formulation, however, as expert elicitation in quantitative risk assessment, for example, also involves expert judgement [137].

The precise classification of TOs and NTOs may also evolve over time. Recently, membership of the *An. gambiae* complex has expanded with the addition of *Anopheles fontenillei* from Central Africa [52]. Moreover, some species of the complex, are currently considered to be only minor vectors of malaria but are still TOs, so that one expected consequence of the intervention could be suppression of species that do not efficiently transmit disease. However, it may still be worthwhile to reduce the density of such vectors, notwithstanding them being inefficient at transmitting disease at present [136].

It could also be argued that many of the pathways identified here, although plausible, would not, in any event, be expected to occur because the potential harm would be identified during standard product development and result in the strain being eliminated from further advancement towards release. There are already regulatory precedents for the development and successful field release of transgenic mosquito strains [20, 138] so that increased vector competence, decreased insecticide sensitivity, broadened tolerances for abiotic conditions, or toxicity and allergenicity of transgenic products are all recognized as having the potential to contribute to harms to health and environmental protection goals. In that respect, many of the pathways identified here are not uniquely drawn from problem formulation. However, this problem formulation approach did help to unpick many of the more complex environmental and ecological pathways to potential harm that need to be further investigated in subsequent steps of ERA for population suppression gene drive. This exercise also allowed for the systematic definition of analysis plans and made the process of identifying and describing potential harms and their plausible pathways demonstrable, transparent and systematic, allowing for any potential for erroneous assumptions or unconscious bias to be challenged.

### Next stages of ERA for population suppression gene drive

This analysis represents, to our knowledge, the first systematic and comprehensive identification of plausible pathways to potential harm using problem formulation for a specific gene drive organism. The exercise involved a systematic approach to identify (a) potential harms and exposure routes; (b) areas of uncertainty in our scientific understanding of plausible pathways to potential harms; and, (c) analysis plans to credibly assess pathways. Although superficially similar to fault tree analyses in some probabilistic risk assessment [137], the purpose of these pathways is not to assign probability to each step, but to inform the next stages of an ERA which will involve exposure and hazard characterization based largely on the outputs of analysis plans identified here. While protection goals were identified from published problem formulation exercises on gene drive conducted in Africa, assessment endpoints for most potential pathways to harm were not precisely defined, nor were limits of concern to those assessment endpoints established. These issues would have to be addressed in the immediate next steps of an ERA for population suppression gene drive.

Depending on the nature of the individual pathway, there are four possible outcomes in subsequent stages of ERA: (i) all hypotheses for a given pathway could be rejected in which case the pathway to harm would be considered valid; (ii) one or more hypotheses that nullify one or more individual steps in a pathway would cause the entire pathway to be rejected; (iii) evidence from a series of hypotheses may together produce sufficient ‘weight of evidence’ to indicate rejection of that pathway; or, (iv) evidence from a series of hypotheses may together produce sufficient weight of evidence to indicate acceptance of that pathway. For example, in Pathway 50, Potential toxicological effects of *dsxF*^*CRISPRh*^ transgenics on NTOs could increase disease transmission in livestock, toxicology tests could be conducted on indicator species and the toxicity of transgenic proteins investigated in the literature and bioinformatically, mostly based on evidence from humans. Nonetheless, evidence from investigation of these evidence requirements should be sufficient to allowed informed decision-making about the potential for such a harm. Additionally, for any given risk hypothesis analysis plans should be tiered so that components likely to contribute most to informing the ERA and allied decision-making can be prioritized [47, 80]. Where a particular pathway is assessed to have the potential to lead to a harm to protection goals following further investigations and characterization of the risk in the ERA, risk mitigation options would need to be subsequently evaluated. It is possible that the unmanaged risk could be considered unacceptable by decision-makers, or they could deem a risk to be acceptable when taking into account the potential benefits of the intervention. It may also be possible to put in place risk management strategies that could be considered sufficiently robust by stakeholders to mitigate any identified risk from the intervention.

## Conclusions

Building on a series of consultative workshops held in Africa that previously identified relevant environmental and health protection goals, eight potentially harmful effects of simulated field releases of the *dsxF*^*CRISPRh*^ transgene in West Africa were identified. These were stratified into 46 plausible pathways that would be required for potential harms to occur. Most identified potential harms involved increased disease transmission, emphasizing the importance to subsequent stages of ERA of data assessing vectorial capacity in transgenics compared to non-transgenics. Although some of the pathways were based on considerations arising from anatomical alterations typically found in *dsxF*^*CRISPRh*^ homozygotes, many pathways could be relevant to field releases of a range of other transgenic strains of mosquito. This analaysis revealed that the efficacy of population suppression caused by the *dsxF*^*CRISPRh*^ transgene should itself have a direct impact on the majority of pathways. Mathematical modelling will therefore play an essential role in subsequent stages of ERA by clarifying the dynamics of this relationship between population suppression and reduction in exposure to specific potential harms [33–35, 139]. This investigation represents, to our knowledge, the first comprehensive identification of plausible pathways to potential harm using problem formulation for a specific gene drive transgene and organism, as well as a transparent communication tool that could inform a full range of future regulatory science studies, guide subsequent stages of ERA [33–35], and stimulate further, broader engagement on the use of population suppression gene drive to control malaria vectors in West Africa.

## Data Availability

Not applicable. All data and material is presented in the manuscript.

## Abbreviations

AUDA-NEPAD: African Union Development Agency (AUDA) New Partnership for Africa’s Development (NEPAD)
ERA: Environmental risk assessment
FNIH: Foundation for the National Institutes of Health
LF: Lymphatic filariasis
NTO: Non-target organism
TO: Target organism
